# Competition between Gas and Liquid Solar Fuel Product Formation from the CO_2_ Reduction Reaction on Two-Dimensional Metal-Organic Framework

**DOI:** 10.1021/acs.jpcc.6c03863

**Published:** 2026-08-06

**Authors:** Aarya D. Riasati, William A. Goddard, Silvio Osella

**Affiliations:** † Materials and Process Simulation Center (MSC), California Institute of Technology, MC 139-74, Pasadena, California 91125, United States; ‡ Materials and Processes Simulation Lab, Centre of New Technologies, University of Warsaw, Banacha 2C, Warsaw 02-097, Poland

## Abstract

Rising CO_2_ emissions in the atmosphere continue to be among the world’s most preeminent environmental challenges. Indeed, the solution of carbon sequestration via geologic storage has proved insufficient to combat this problem. The alternative to sequestration is the electrochemical reduction of carbon dioxide to liquid products, a common step to combat net warming effects. However, the electrochemical reduction of carbon dioxide remains limited because of several competing pathways and potential-dependent kinetics at catalytic surfaces. Herein, we consider Cu-phthalocyanine Metal–Organic Framework (MOF) as a candidate electrocatalyst to reduce CO_2_ to a single hydrocarbon product. To achieve this, we develop a first-principles Grand Canonical Potential Kinetics (GCP-K) framework for quantifying the activity and product selectivity of said MOF catalyst. Our model is derived directly from constant-potential DFT calculations, from which reaction free energies and activation barriers are extracted across the relevant electrochemical manifold. Potential dependence is incorporated through explicit charge-transfer symmetry factors, enabling the systematic construction of potential-resolved rate constants. On this basis, we assemble a microkinetic reaction network that encompasses the competing pathways leading to C1 and C2 liquid products. We calculate Faradaic efficiency maps across multiple selectivity windows from branch points in different microenvironments. We find that charge-transfer processes shift the adsorption energy of any individual intermediate by only a few millivolts per 0.1 V of applied bias, but the cumulative effect across multistep PCET sequences displaces the selectivity windows by tens to hundreds of millivolts. We calculate selectivity-limiting steps and the various kinetic rates associated with them as a function of potential and surface coverage. In addition to mechanistic investigations of each reaction framework in question, we elucidate the stabilization of various oxygenated intermediates and their role in creating different C1 and C2 products. Taken as a whole, these results show a variety of catalyst-level design levers that could increase the liquid product window and offer a mechanistic foundation for selecting the operating potentials that maximize desired product selectivity at functional current densities.

## Graphical abstract

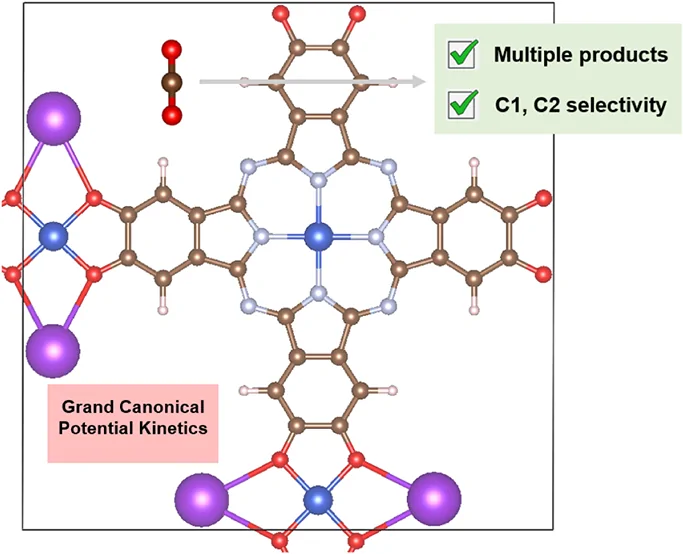

## Introduction

Among the world’s most prevalent challenges are the rising CO_2_ emissions in the atmosphere. Mitigating anthropogenic CO_2_ emissions while meeting a rising global demand for fuels and chemicals ranks among the grand challenges of contemporary catalysis. In this regard, net carbon sequestration via geologic storage has proven effective as a safe and permanent practice, but conversion to fuel synthesis is far more appealing in regards to reducing emissions.^1^ In principle, electroreduction of carbon dioxide offers a compelling path forward because it can couple the overall carbon cycle to renewable energy and close the loop on energy and carbon.^2^ However, even with the potential upsides of electrochemical coupling, industrial deployment has stalled.

First, even the best Cu electrodes suffer from poor selectivity: generating a spectrum of C1 and C2 hydrocarbons and oxygenates along with CO and H_2_, undermining process economics.^3^ In addition, most state-of-the-art electrochemical carbon dioxide reduction reaction (CO_2_RR) catalysts rely on precious or heavy metals (silver, gold, palladium) that are expensive, scarce, and environmentally problematic. It thus remains an urgent scientific and technological priority to find earth-abundant, metal-lean catalysts that can deliver a single value-added product at high current density.

An alternative approach, two-dimensional metal–organic frameworks (2D-MOFs) have emerged over the past years as a versatile platform for heterogeneous electrocatalysis that may address both concerns. The atomically thin, π-conjugated sheets of 2D-MOFs offer high active-site exposure, tunable electronic structure, and the ability to position catalytically relevant heteroatoms (N, B, P, S) in precise coordination environments to tune these sites for maximum efficacy,^4^ without the weight penalty of bulk metals. These MOFs compounds can provide a fully organic backbone to chelate transition metals or else remain metal free. This can convert the framework to a “single-atom” catalyst effectively modulated by the electronics of the ligand substituents as opposed to the metallic bands involved in a bulk metal.^5^ Prime examples of such substituents include porphyrin or phthalocyanine motifs.^6,7^ In aqueous electrolytes such 2D sheets exfoliate spontaneously, creating large interfacial areas while preserving their structural order. Here their in-plane pores serve as molecular sieves that modulate mass transport and enforce shape-selectivity unattainable on close-packed metal surfaces.^8^

Among the portfolio of solar-fuel targets, lower alcohols occupy a vantage position. Methanol is already traded at the scale of 100 Mt/yr as a platform molecule for formaldehyde, acetic acid, and olefins, whereas ethanol exceeds 120 Mt/yr while doubling as a high-octane blending agent in gasoline.^9,10^ These two alcohols can successfully be fed directly into the existing infrastructure as is, effectively avoiding handling problems that plague gaseous products. This makes these alcohols much more attractive to ventures involving renewable energy and electricity.^11^ Consequently, a catalyst that channels CO_2_ selectively into methanol or ethanol under mild conditions would enable distributed fuel and chemical synthesis with minimal additional engineering.

Here, we build on these themes by interrogating a phthalocyanine-based (2D-MOFs) recently reported that rivals Cu in forming both C1 and C2 gas products, albeit with distinct kinetics.^12^ Our prior computational studies have suggested that this 2D-MOF favors direct coupling of *CO with *CHO to form C2 products, bypassing the classical rate-determining *CO–*CO dimerization step observed on metallic copper. This could influence the distribution between oxygenates and hydrocarbons at moderate overpotentials.^12^ To dissect the atomistic level mechanism for these processes, we deploy Grand-Canonical Potential Kinetics (GCP-K), a density-functional theory (DFT) framework in which the applied electrode potential is treated as the thermodynamic variable on equal footing with atomic coordinates. This GCP-K approach avoids the inconsistency in constant-charge simulations. It yields potential-dependent reaction free energies and activation barriers that flow naturally into microkinetic models.^13^

We summarize here our three principal findings.

i In the C1 manifold, the phthalocyanine 2D-MOF prefers methanol over methane, despite the latter’s deeper thermodynamic well, because the alcohol branch avoids a 0.5 eV activation barrier associated with *CHO to *CHOH hydrogenation.
ii For C2 products, we find that both vinyl- and glyoxal-centered coupling paths coexist. We observe that vinyl dominates at moderate bias leading to ethylene, whereas glyoxal emerges at more negative cathodic potentials tipping selectivity toward ethanol once the hydrogenation barrier to *­(CHO)­CH_2_OH goes below 0.26 eV.
iii The catalyst single-atom active site confers fragment-sensitive kinetics that differ substantially from those on metallic copper. This can be attributed to ligand electronic effects, where instead of *CO dimerization, the surface population of *CHO and *CCHOH fragments becomes the master switch between gas- and liquid-phase products.

By unraveling these mechanistic pathways, we rationalize hitherto puzzling experimental trends while furnishing a design roadmap for next-generation 2D-MOF electrocatalysts. We believe that the methodology and insights presented here will accelerate the transition from metal-rich, poorly selective CO_2_RR electrodes to earth-abundant, alcohol-specific 2D catalysts compatible with the circular-carbon economy.

## Methods

The methodology used in this paper has been validated in our previous study on the formation of methane and ethylene from the same MOF catalytic center.^12^ The same settings to generate the periodic unit cell, its dimension and parameters to perform the calculations were adopted for the current study. In brief, spin-polarized density functional theory (DFT) on periodic 2D cells as implemented in the Vienna ab initio simulation package (VASP) was used for geometry optimization and phonon calculations.

To take into account the role of water in the electrochemical cell, we used VASPsol to include implicit solvation.^14–18^ In addition, a cluster of four water molecules was explicitly added to the system to accurately describe the proton transfer from the solvent to the adsorbate (Figure S1 in Supporting Information). In order to obtain a better description of the solvent effect, and to account for the applied potential, after geometry optimization of the system with VASP, we performed a single point calculation using JDFTx and the CANDLE (Coupled-Atom Neighbor Density–Localized Electrostatics) description of the implicit solvent.^19,20^

At this stage of the computational protocol, after obtaining the Gibbs free energy for the different intermediates, we applied the Legendre transformation to obtain the grand canonical potential kinetic energy GCP-K.^21–23^ In this approach, the applied potential is kept constant during the electrocatalytic reactions, while the charge near the active site is allowed to change along the reaction pathway. Thus, the local charge is different from the reactant, the transition state, and the product but the applied potential is the same. The GCP-K approach greatly simplifies this procedure, making it very efficient. Briefly, the total free energy as a function of the number of electrons, F­(n), is obtained for each reaction step using standard QM as a function of the net charge. Then, a Legendre transformation to convert F­(n) to the grand canonical free energy, G­(n; U) is considered:

1 $$G\left(n;\;U\right)=F\left(n\right)-ne\left(U_{SHE}-U\right)$$

where the grand canonical free energy *G* depends on the number of electron (*n*) and the applied potential (*U* vs SHE) and U_SHE_ = μ_e,SHE_/e is the electronic energy at standard hydrogen electrode (SHE) condition. The complete derivation of the formalism can be found elsewhere.^17,18^ The power, efficiency and accuracy of the GCP-K method has been validated for semiconductors in our previous work.^12^

The Brønsted–Evans–Polanyi (BEP) relationship was considered to compute the activation energies^24,25^ for electrochemical reactions.^26^ In this approach the transition state for the nonelectrochemical step is equivalent to that of the electrochemical step at a specific potential *U*:

2 $$\Delta G^{act}\left(U\right)=\alpha \left(U\right)\left(\Delta G\;\left(U\right)+\mathrm{neU}\right)+\beta \left(U\right)$$

where α­(*U*) and β­(*U*) represent the best fit slope and intercept of the BEP scaling relation that varies with potential. Thus, the free energy at the fixed potential, say −1.2 V, is obtained with the GCP-K method. The α parameter is a potential-independent constant that is typically between 0.3 < α < 0.7. We find *α =* 0.456, which is close to the commonly used value of 0.5, and β = 0.36. As a result, we can predict with good accuracy the potential-dependent activation energy (Δ*G* ^*act*^ (*U*)) for each elementary reaction, without the need to conduct expensive, time-consuming, and computationally demanding DFT barrier calculations.^8,27^ Because the C1 selectivity between methanol and methane is set by the difference between two proton-coupled activation barriers at the shared *CHO branch point, both of which scale with their reaction free energy through the same BEP relation, this barrier difference, and hence the C1 branching ratio, is essentially independent of the precise value of α across the physically accepted range 0.3 < α < 0.7 (see Table S7 in Supporting Information). Beyond the α-dependence, uncertainties associated with BEP activation energies can be related to the exchange-correlation functional used, as well as the description of the electrolyte. Because the selectivity mainly rests on the difference between barriers of different and competing elementary steps at different branch points, and competing steps are present at the same active sites, solvation networks, and BEP relation, systematic errors largely cancel. As a result, the rate-determining steps do not change, and, in the case of ethylene formation where the individual split is indeed the smallest between 0.27 and 0.34 eV, assignment is not based on that difference, rather on the cross-path eliminations that are barrierless.

All energies reported in this study are Gibbs free energies in eV at 298 K, which includes zero-point energy, the temperature contribution to enthalpy, and entropy. Reaction mechanisms are described at a fixed applied bias of −1.2 V, and all values reported are grand-canonical free-energy differences where each proton–electron transfer is referenced to the explicit H_9_O_4_^+^/4H_2_O cluster. For steps in which a preceding open-shell intermediate is converted to a closed-shell intermediate, the Cu–C distances increase from ∼1.9 Å to ∼3.40 Å (see Table S1). Thus, radical-quenching hydrogenations release most of the newly formed C–H bond energy, leading to exergonicities of several eV at strongly cathodic bias, which are a physically expected outcome. Neutral pH is considered throughout the study. pH enters the grand-canonical formalism explicitly through the proton chemical potential, which shifts as −2.303 *k*_B_ *T* × pH; consequently, the onset potentials of products whose rate-determining step is proton-coupled shift Nernstially, by ≈59 mV per pH unit on the SHE scale, whereas steps that are chemical (nonelectrochemical) in nature are unaffected, so that the relative ordering of the products and the qualitative structure of the selectivity windows are preserved across pH.

This study analyzes 57 elementary surface reactions, each evaluated at seven different electrode potentials ranging from −0.80 to −2.00 V versus the Standard Hydrogen Electrode (SHE). For each potential microkinetic analysis was performed, considering intermediate and transition structures as described above, and referenced to gas-phase CO_2_ + 4 H_2_O. The forward and reverse activation energies are obtained from the BEP method. Negative values delineate barrierless steps at the specified potential, by which we mean that the BEP-extrapolated activation free energy lies at or below the thermal energy (*k*_B_ *T* ≈ 0.026 eV at 298 K) rather than being identically zero; such values arise for the strongly exergonic hydrogenation and water-elimination steps and denote processes that proceed much faster than the rate-determining steps of the network, with potential-resolved forward and reverse rate constants calculated using harmonic transition state theory at room temperature with the pre-exponential factor of 10^13^ s^–1^ and a Boltzmann factor of k_B_ = 8.617 × 10^–5^ eV K^–1^. The resulting *k*_f_/*k*_b_ matrices are then feed into a stiff Livermore Solver for Ordinary Differential Equations with Automatic method switching (LSODE) integrator to solve the mean-field site-balance ordinary differential equation (ODE) system at a surface-site density of 2.40 × 10^–9^ mol cm^–2^, which is calculated from the real-life orientation of the system in question. Convergence was declared when the net flux at every node fell below 1 × 10^–1 2^ mol cm^–2^ s^–1^.

## Results and Discussion

In this paper, we elucidate the mechanisms to form different solar fuels products, including multistep reductions to obt a variety of different C1 and C2 products on the selected 2D-MOF. Briefly, the key proton-coupled electron transfer (PCET) steps to form the different products are

- 4e^–^ to obtain CH_2_O (formaldehyde),
- 6e^–^ to obtain CH_3_OH (methanol) or CHOCHO (glyoxal),
- 8e^–^ to obtain CH_4_ (methane) or CHOCH_2_OH (glycolaldehyde),
- 10e^–^ to obtain CHCH (acetylene), CH_2_CHOH (vinyl alcohol), CH_3_CHO (acetaldehyde) or (CH_2_OH)_2_ (ethylene glycol),
- 12e^–^ to obtain CH_3_CH_2_OH (ethanol) and C_2_H_4_ (ethylene).

The large number of different possible gaseous and liquid products that can be formed from the reaction leads to poor selectivity. Moreover, the sluggish kinetics and low solubility of CO_2_ also contribute to the low yields obtained. In addition, the hydrogen reduction process can strongly compete with the CO_2_RR, further decreasing the product yields.

Here, we report a series of Quantum Mechanics (QM) calculations of a square phthalocyanine-based 2D-MOF that can be considered as single atom catalyst, with the copper atom bound to the phthalocyanine acting as catalytic center. In our recent publication, we showed that this catalyst can form gas phase ethylene in high yield and selectivity, but we did not analyze the kinetics of the process nor the formation of liquid phase products, which is the focus of the current study.^12^ We assess the mechanism for formation of liquid products, either C1 such as methanol or C2 products including ethanol, acetaldehyde, vinyl alcohol, glyoxal, glycolaldehyde and ethylene glycol, which are accessible through various shared formation pathways (Scheme 1). These products would remain in the water-based solvent and have not yet been reported experimentally. Moreover, we performed microkinetic analysis in the −2.0/–0.8 V bias range, to assess the selectivity of forming the different products, their turnover frequencies, and generated current, providing a complete description of this complex reaction.

**Scheme 1:**
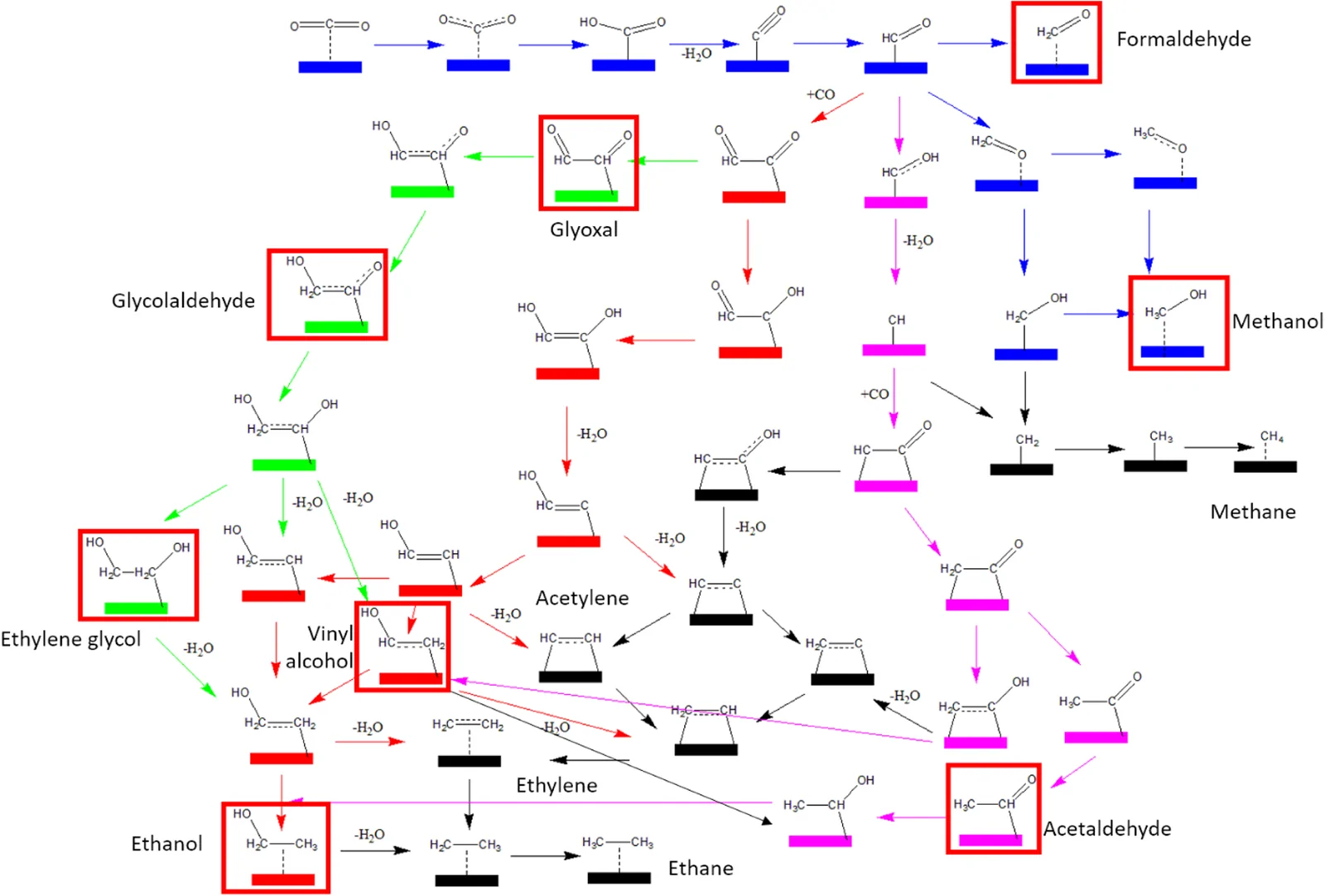
Predicted Reaction Pathways for CO_2_ Reduction to Various Gas Phase and Liquid Phase C1 and C2 Products^a^

a The focus of this paper is the formation of liquid products such as methanol (blue path), ethanol (green and red paths) as well as the possible different intermediates (labelled and indicated with red boxes).

### Formation of C1 Products

The first part of the reaction path toward the formation of methanol is shared with the methane path, up to the formation of the *CHO intermediate. From Figure 1, we see that the entry toll to the C1 network is up from *CO, where the first PCET establishes both the thermodynamic slope and the dominant kinetic hurdle (Figure 1a). From here, and starting from CO_2_ in the gas-phase, the surface becomes downhill, (ΔG = −0.28 eV) with a small entrance barrier of about 0.23 eV (Figure 1b). The subsequent PCET to form *HOCO constitutes the universal early bottleneck for all C1 outcomes: at −1.20 V its activation free energy is 0.42 eV and, in the Brønsted-Evans–Polanyi (BEP) treatment, this barrier exhibits only a weak potential sensitivity, drifting slightly with cathodic bias but never collapsing. Once *HOCO is formed, the decarboxylation to *CO is barrierless and strongly exergonic (ΔG = −2.14 eV). The subsequent hydrogenation to form *CHO from *CO then proceeds readily with an activation barrier of 0.18 eV and ΔG = −0.38 eV.

**Figure 1:**
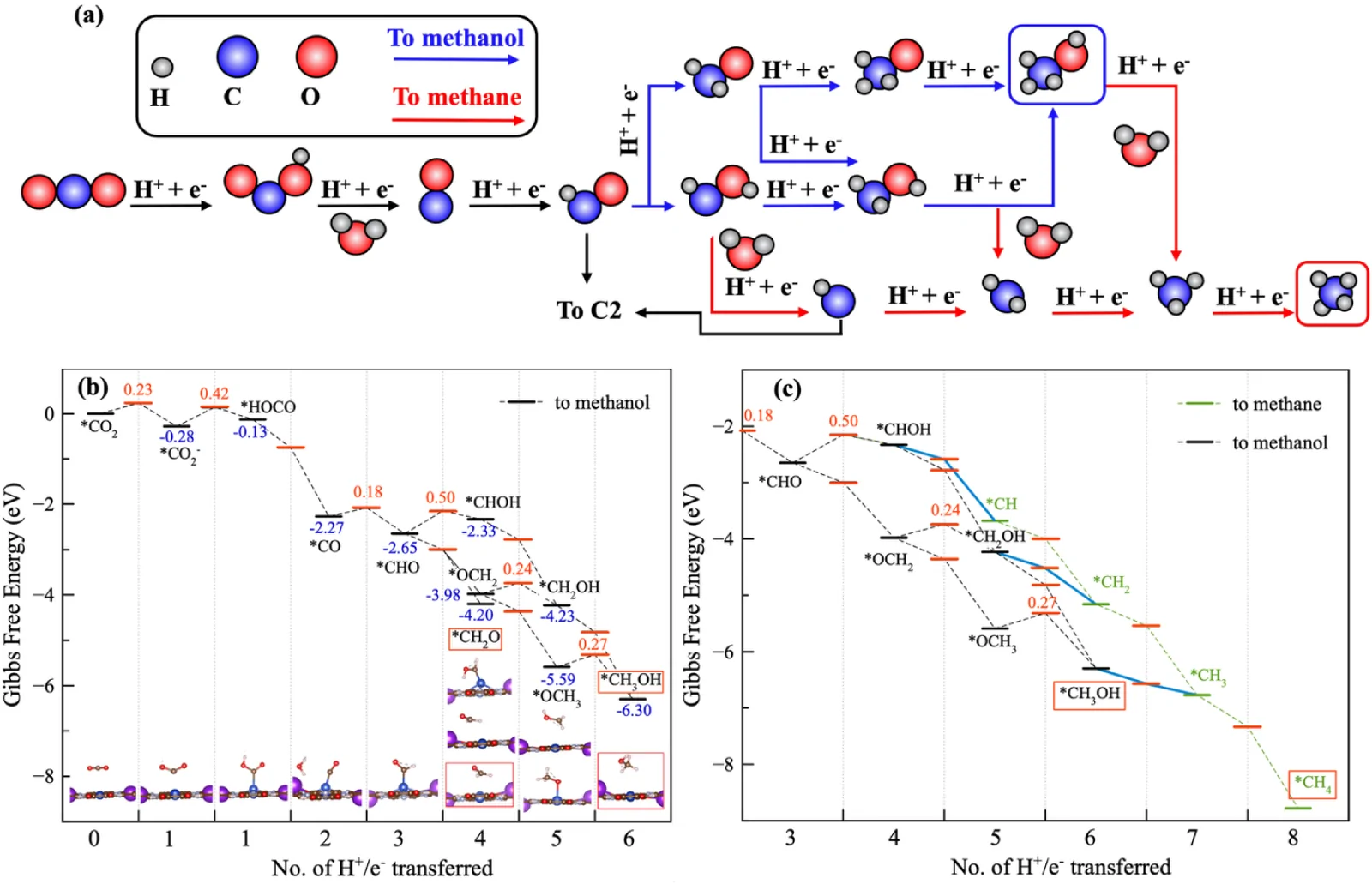
(a) Reaction network for the C1 branch of CO_2_ electroreduction on the MOF active site. Individual intermediates are also depicted (red = O, blue = C, white = H); blue arrows trace the kinetically favored path to liquid methanol, and red arrows illustrate the competing path to gaseous methane. (b) Gibbs free-energy profile computed at ambient conditions (298 K) at V = −1.2 V. The dashed black curve follows successive PCETs leading to methanol, which are shown in potential energy wells. The red segments refer to the transition states along those same paths. (c) Gibbs free-energy competition profile between methanol and methane obtained via the GCP-K protocol described above. The thick cyan lines refer to water elimination steps, where the methanol and methane paths cross overall.

For the formation of *CHO, there are two main steps that drive the reaction, the modest barrier for bending, and then the key hydrogenation step as the main branching mode. We see that at *CHO, the manifold twists into four different C1 routes with differing fluxes based on the ease of the first step. CO is maintained as only a sink at different and small biases, simply because the rate to form *CHO is downhill and fast. At this point, desorption cannot compete once the main PCETs accelerate, with the result that CO contributes only a small part of the Faradaic Efficiency (FE) map.

From Figure 1, we see that formaldehyde emerges as a transient C1 product rather than a terminal C1 product: carbon-centered formation of *CH_2_O from *CHO proceeds with ΔG = −1.55 eV, with the next step to form *OCH_3_ also barrierless with similar thermodynamics. Because both steps are relatively activation-barrier free, *CH_2_O does not accumulate readily until/unless the electrochemical bias is shallow, appearing only at very low overpotentials. *CH_2_O once formed leads to methanol as the dominant process, but with the formation of *OCH_3_ from the initial formaldehyde step at low biases.

Methanol formation proceeds through two convergent channels, of which the methoxy route is dominant across the potentials evaluated. The formation of *CH_2_O from *CHO is barrierless, with the overall formation of CH_3_OH barrierless except in the last step of *OCH_3_ to CH_3_OH, which presents a small barrier (Ea) of 0.27 eV. Thus, for the C1 mechanism, the rate-determining step (RDS) for the overall process remains *HOCO formation. After the initial formation of *CO, all additional steps are essentially barrierless. The hydroxyl route supplements this behavior: *CH_2_O → *CH_2_OH has a barrier of only 0.24 eV and is nearly isoenergetic (ΔG = −0.03 eV), while *CH_2_OH → CH_3_OH is barrierless and strongly downhill (ΔG = −2.07 eV). This auxiliary channel does not alter the identity of the RDS but broadens the bias range over which methanol dominates by siphoning any *CH_2_O in the reservoir toward alcohol rather than allowing it to desorb or back-react.

In contrast, methane product is throttled through the conversion of *CHOH from *CHO (Figure 1c). At −1.20 V this step has Ea = 0.50 eV and is thermodynamically uphill (ΔG = +0.32 eV). Beyond this point, the mechanism then falls down the thermodynamic hill through mainly barrierless steps to full hydrogenation (*CHOH → *CH → *CH_2_ → *CH_3_ → *CH_4_). With increasing negative potential, the *CHO → *CHOH barrier relaxes (to ∼0.33 eV by −2.0 V), but it remains higher than the 0.27 eV capstone to CH_3_OH, preserving methanol’s kinetic edge over a broad window.

### Competition with Gas Products: Methanol vs Methane

At the benchmark bias of −1.20 V the grand-canonical landscape unequivocally favors the kinetic route to methanol over that to methane, even though the latter is thermodynamically more exothermic by ΔG = 2.48 eV. Overall, the methane pathway is strongly unfavorable. However, a cross-path from methanol to methane is present, with three possible crossing water elimination steps: *CHOH → *CH, CH_2_OH → *CH_2_ and CH_3_OH → *CH_3_, all of which are barrier free and exergonic, rationalizing the experimentally observed methane formation (Figure 1c). From Figure 2a, we see that the competition between liquid methanol and methane is selective as a result of the first steps from the first *CHO intermediate. The hydroxyl route to form liquid methanol is the dominant C1 outlet at 10 mA cm^–2^, while the methoxy route is negligible due to the formaldehyde formation. Methane production is very similar in magnitude at each respective bias, and strongly decreases particularly between −1.6 V and −1.2 V, where *CHOH suppresses the formation of CH_4_.

**Figure 2:**
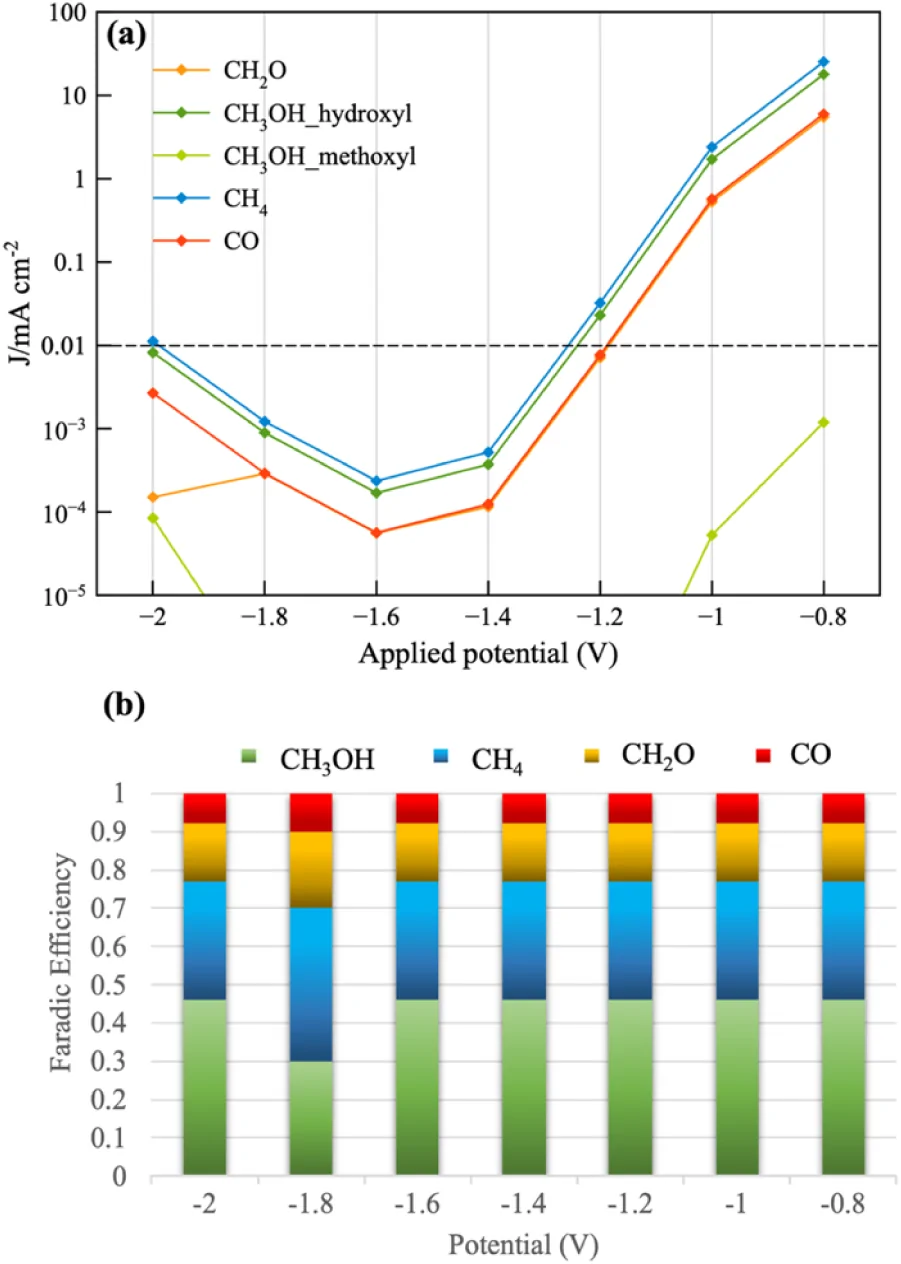
(a) Potential-dependent partial current densities for C1 products, over the range from −2.0 to −0.8 V. The black dotted line is the 10 mA/cm^–2^ reference. (b) Product selectivity for the C1 product manifold, expressed as the overall fractional Faradaic output for CO, CH_2_O, CH_4_, and CH_3_OH. The Faradaic efficiency for methanol exceeds that for methane at every bias in this range. The Faradaic efficiencies in this panel, and in the corresponding panels for the C2 manifolds (Figures 5b, 6b and 7b), are normalized within each reaction manifold and therefore report intrabranch selectivity; a global Faradaic efficiency summed over all C1 and C2 carbon products at each potential can be reconstructed directly from the rate-constant matrices tabulated in Tables S3–S6.

We see that CO and CH_2_O production become prominent only at −1.0 V and −0.8 V, which is consistent with fast *CO hydrogenation and overall weak binding of formaldehyde. The CH_2_O channel is reduced once the two methanol channels accelerate, converting these qualitative observations to quantitative estimates (Figure 2b). At every potential shown, the combined methanol fraction exceeds the methane fraction. For example, at −1.2 V we find 23–25% on the methoxy route and 20–22% on the hydroxyl route, summing up to 45–47% FE, while methane gives a lower 30% yield. Both formaldehyde (15%) and CO (5% FE) are minor products, even at −2.0 V, where the *CHOH barrier has relaxed and the CH_4_ band expands. Still, the methanol product leads methane. Conversely, toward −1.0 → −0.8 V all C1 currents compress by ∼1 to 3 orders of magnitude, becoming more positive. Here CH_2_O and CO gain relative weight but their absolute current remains small, while the methanol advantage in current persists because its entry channel has a small barrier and methane does form despite the high formation penalty for *CHOH from *CHO.

Overall, we see that the barrier of CO_2_ to *HOCO dominates the activity of the C1 product scheme, while also determining the products being formed. We also observe that the methanol manifold exploits barrier-free hydrogenations until the final 0.27 eV step, before once again exhibiting a thermodynamic downhill fall to form the final product. In contrast, the formation of methane must first surmount a larger 0.5 eV activation barrier to form *CHOH, suppressing its formation. CO and formaldehyde form at low potentials of −0.8 V and −1.0 V with fast, barrierless steps leading to the formation of diverse products in the C1 scheme toward ultimate methanol formation and away from methane.

### C2 Product Formation: The Pathways to Ethanol and Other Liquid C2 Products

As reported in our recent publication,^12^ three distinct C–C-coupling topologies emerge from kinetic analysis, two of which are obtained from the CHO–CO coupling and one from the CH–CO coupling. These are labeled “vinyl-alcohol”, “glyoxal” and “acetaldehyde” paths, respectively, and each of them funnels into a common late-stage hydrogenation sequence that terminates with ethanol (Figure 3).

**Figure 3:**
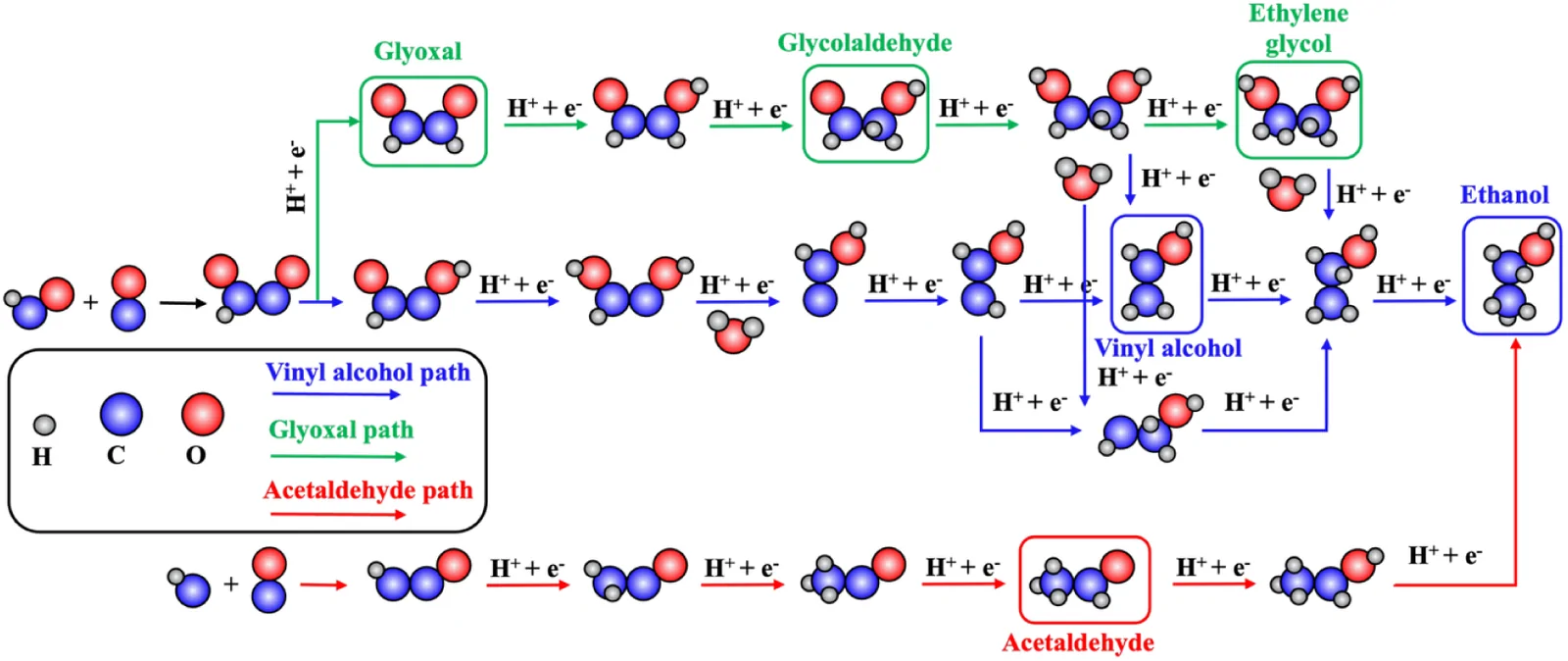
Reaction map for the creation of C2 products on the phthalocyanine-based 2D-MOF. We see that there are three competing manifolds resulting in the same product: liquid ethanol. The blue “vinyl-alcohol” path moves through vinyl alcohol before final hydrogenation to ethanol. The green “glyoxal path” branches off via dimer-derived *­(CHO)­CO fragments and passes successively through glyoxal, glycolaldehyde, and ethylene glycol. Finally, the red “acetaldehyde path” moves through acetaldehyde before stopping at the hydrogenation stage. Adsorbed intermediates are also depicted (red = O, blue = C, white = H).

In the vinyl-alcohol path (Figure 4a), the association of *CO and *CHO into the ketenyl adduct *­(CHO)­CO is remarkably barrierless, strongly favoring its formation from both kinetic and thermodynamic perspectives. The first step is vinylization, hydrogenating the dimer oxygen and carbon in series to *­(CHO)­COH and *­(CHOH)­COH. The PCET from this step leads to the highest barrier on this branch with an activation barrier of 0.28 eV. The pathway then bifurcates after dehydration to *CCHOH and the carbon-side hydrogenation to *CHCHOH proceeds downhill. There are no additional positive barriers in the vinyl-alcohol leg, which is favored thermodynamically, and followed by subsequent hydrogenation into the β-hydroxy-ethyl intermediate (*CH_2_CH_2_OH) that is strongly exergonic. Then, the final step to ethanol is also barrierless, confirming both the thermodynamic and kinetic preference toward its formation.

**Figure 4:**
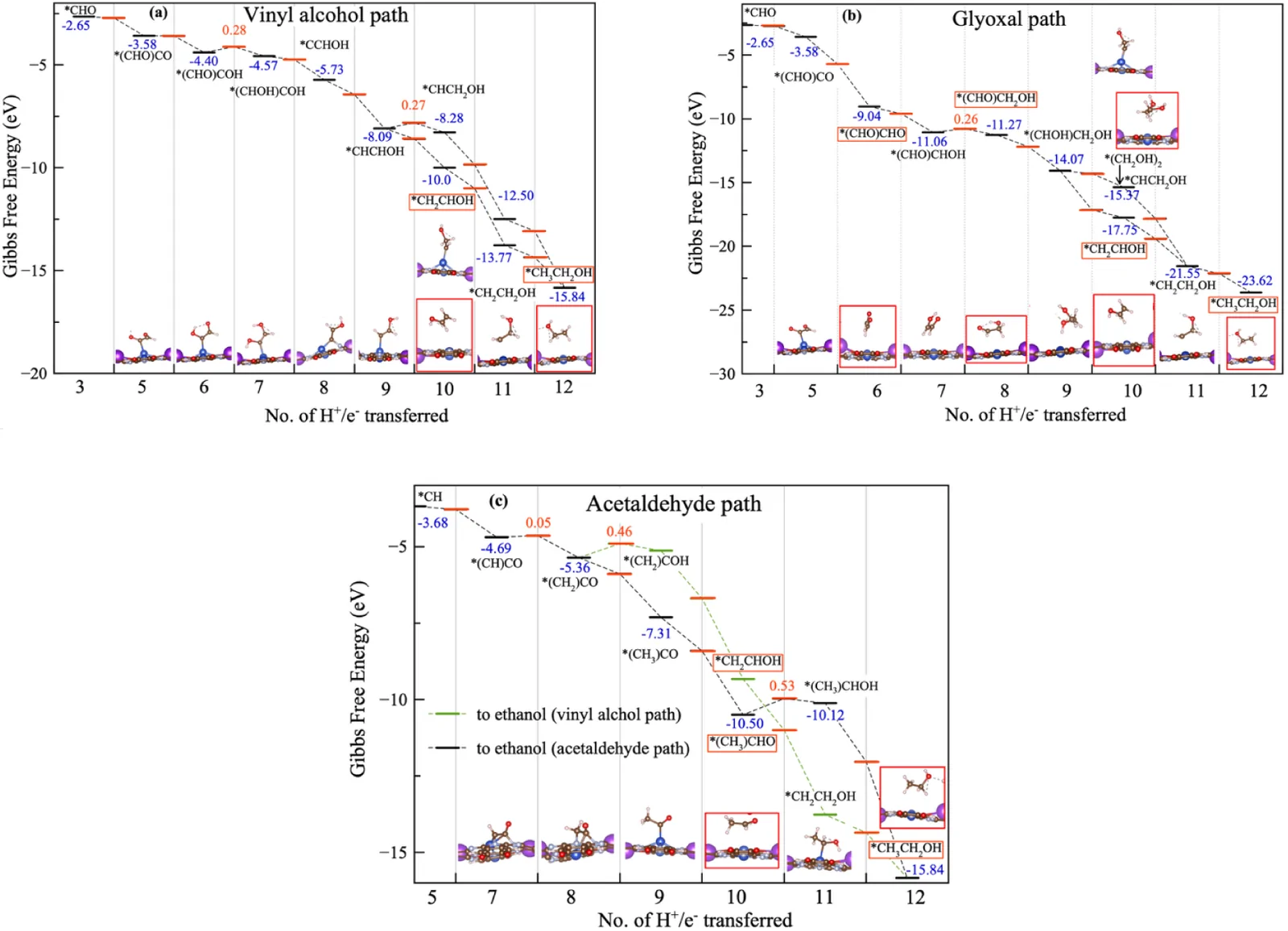
Grand-canonical free-energy profile at neutral pH, a bias of −1.20 V and at 298 K, for (a) vinyl-alcohol (b) glyoxal and (c) acetaldehyde coupling pathways. The dashed black curve illustrates several PCETs that lead to ethanol, whereas the red lines depict the transition states in the overall potential energy diagram. Labels give the activation barriers and reaction energies for each step as well, along with the geometries of each adsorbed intermediate.

The second leg of the bifurcation forms *CHCH_2_OH, which requires an activation barrier of 0.27 eV, followed by a barrierless cascade toward the formation of ethanol.

We see that the glyoxal path (Figure 4b) diverges from the same *­(CHO)­CO node but now protonates the two carbonyl centers alternately, leading to different liquid products.

The first carbon-side PCET to *­(CHO)­CHO (glyoxal) is very exergonic with ΔG values of −5 eV (see Methods for the referencing free energies), followed by an oxygen-side PCET to *­(CHO)­CHOH that remains downhill.

The second carbon-side PCET to *­(CHO)­CH_2_OH (glycolaldehyde) introduces the largest span on this branch with an Ea = 0.26 eV and is only slightly downhill in free energy, which is why glycolaldehyde accumulates. From there, two scenarios are possible: additional oxygen protonation toward *­(CHOH)­CH_2_OH and ethylene-glycol formation ((CH_2_OH)_2_) through another C-side PCET or dehydration to vinyl alcohol (or *CHCH_2_OH) and reconvergence with the vinyl funnel. Both of these pathways are barrierless. Because each oxygen-retaining step is downhill and essentially activation-free, the glyoxal topology naturally pools flux in oxygenated liquids (glyoxal, glycolaldehyde, ethylene glycol), explaining the elevated FE of polyols and the lower ethanol current for this branch at a given potential.

In the acetaldehyde path (Figure 4c), *CH couples with *CO to give an acetyl intermediate via a barrierless association (*CH/*CO → *­(CH)­CO). The first hydrogenation to obtain (CH_2_)­CO only requires a minimal activation barrier of 0.05 eV, and a strong stabilization of −0.64 eV. Furthermore, additional hydrogenation of the methylene carbon to *­(CH_3_)­CO is barrierless and strongly downhill, with ΔG values less than −2 eV, leading to the facile formation of acetaldehyde *­(CH_3_)­CHO after another barrierless downhill step where ΔG is around −3 eV. This step is followed by an endergonic, direct hydrogenation of acetaldehyde to *­(CH_3_)­CHOH, having the largest activation barrier of 0.53 eV and eventually leading to ethanol. This path exposes a bifurcation that restrain the overall throughput of the reaction, the second of which is protonation of the oxygen to form *­(CH_2_)­COH from *­(CH_2_)­CO. This step requires to overcome an energy barrier of 0.46 eV and is as a result uphill, contributing meaningfully to ehtanol formation only at very negative potentials. Because of this, its internal FE is ethanol-dominant with acetaldehyde as a recurring yet persistent coproduct.

We observe that in the vinyl-alcohol path, early association to *­(CHO)­CO is strongly exothermic and barrierless, while the first kinetically demanding conversion from the vinyl to allyl intermediate occurs with a modest, finite barrier Ea= 0.28 eV. Following this, we observe that hydrogenation into the β-hydroxy-ethyl intermediate (*CH_2_CH_2_OH) is highly favorable. Terminal hydrogenation to ethanol is weakly activated too, producing an overall pathway that is primarily controlled by the ketenyl/vinyl step. The potential-resolved currents (Figure 5a) corroborate this with the *CH_2_CH_2_ intermediate carrying the lowest current within the vinyl set, the vinyl *CHCH intermediate and the β-hydroxy-vinyl *CH_2_CHOH a few-fold higher, and the final ethanol current higher than for the *CH_2_CH_2_ path.

**Figure 5:**
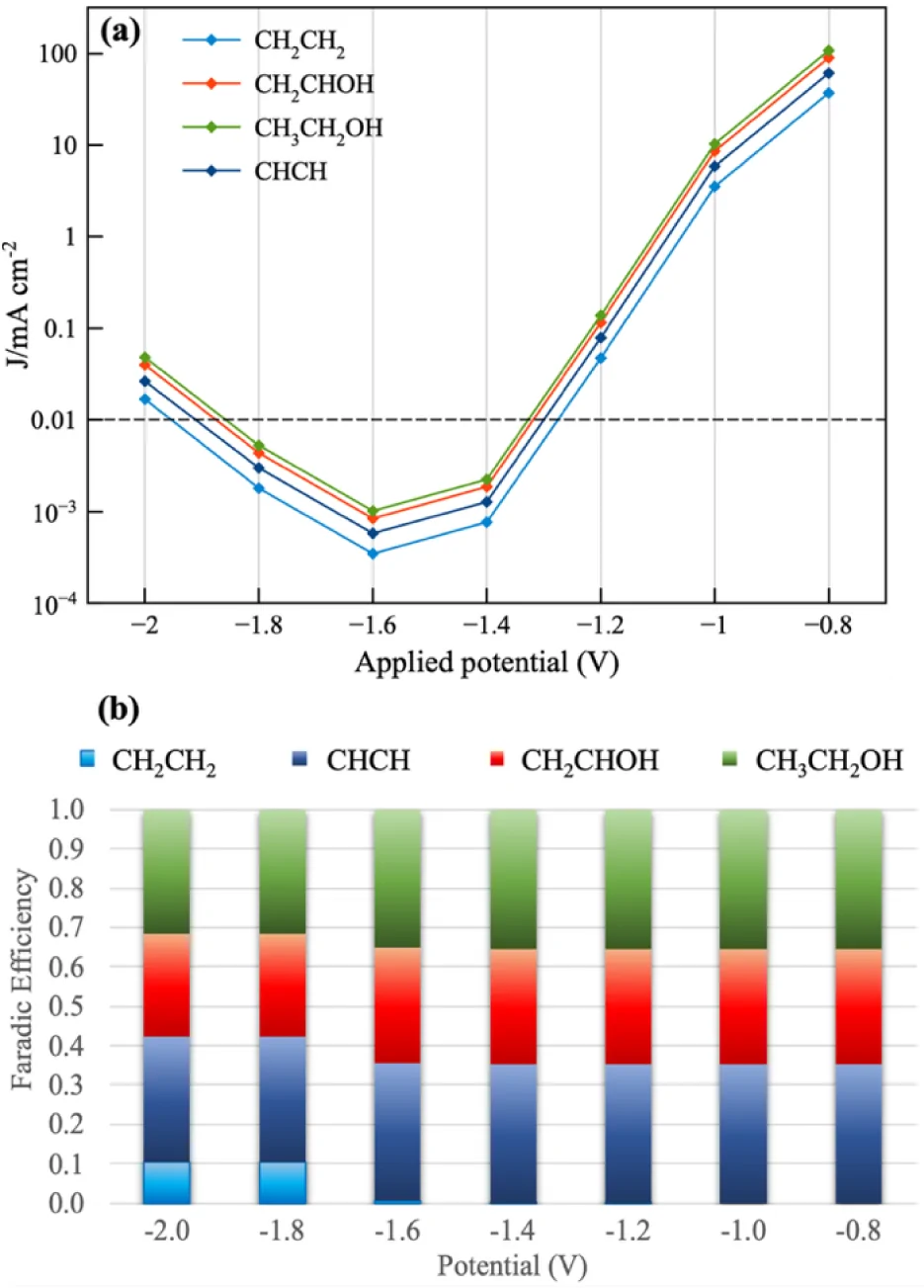
(a) Potential-dependent partial current densities for C2 products from the vinyl path (−2.0 to −0.8 V). The black dotted line is the 10 mA/cm^–2^ reference. (b) Faradaic efficiencies within the vinyl path.

Notably, all four products increase in parallel toward both deeper (≥|−1.8| V) and milder (≤|−1.2| V) bias, signaling that coverage of the coupled vinyl reservoir rather than a late barrier sets the flux. In Faradaic terms (Figure 5b), ethanol dominates internally across the entire window yielding 35–40% FE, whereas the ethylene-leaning fragments (*CH_2_CH_2_ + *CHCH) together hold 30–35% FE, increasing slightly at the most negative points as hydrogenation barriers decrease. The *CH_2_CHOH part remains minor, reflecting its rapid conversion to *CH_2_CH_2_OH. These vinyl-manifold fingerprints explain why ethylene emerges first at moderate overpotentials (its precursor channel is favored) yet ethanol catches up as the final hydrogenation becomes easier and *CH_2_CH_2_OH formation increases in the global C_2_ pathways for multiple product formations.

The glyoxal path takes the same dimer core *­(CHO)­CO but shunts it through sequential carbonyl protonations that stabilize deep oxygenated intermediates before reconverging either via vinyl alcohol or the β-hydroxy-ethyl pathways toward ethanol. The applied current potential curves show glycolaldehyde and ethylene glycol with seemingly identical shapes and magnitudes, and very weak dependence on the applied and final potential (Figure 6a). For example, for ethanol, the resulting current is highest among all the different products. Overall flux in the scheme is detained upstream in the oxygenate basin, resulting in ethanol carrying the highest output and current. FE analyses (Figure 6b), makes this effect quite clear: the FE of ethylene glycol and glyoxal together yields 50–55% FE across the entire window, while ethanol yields a relatively smaller 30%, and glycolaldehyde remaining below 10%. Application of deeper cathodic biases increases the overall fraction of ethylene, but only minimally.

**Figure 6:**
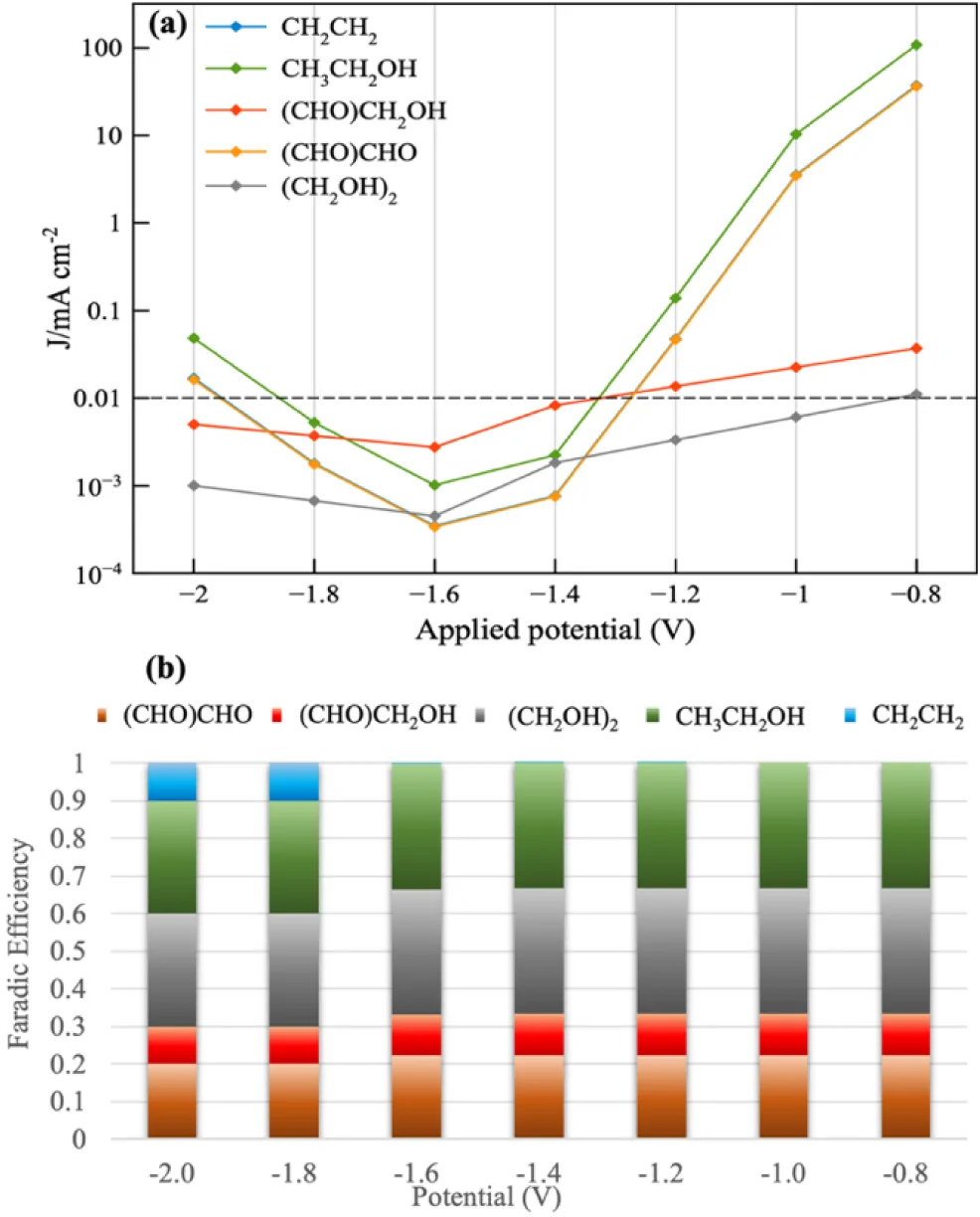
(a) Potential-dependent partial current densities for C2 products from the glyoxal path (−2.0 to −0.8 V). The dotted black line is the 10 mA/cm^–2^ reference. (b) Faradaic efficiencies within the glyoxal topology, presented above.

In the acetaldehyde path, the C1 hydrogenation product of *CH couples with *CO to give an acetyl intermediate. Of the three paths, the protonation of the oxygenation for conversion of *­(CH_2_)­CO to *­(CH_2_)­COH (and eventually to vinyl alcohol) carries the largest activation energy among the three different pathways investigated. Thus, this pathway is suppressed at moderate bias. The applied current potential plot (Figure 7a) reveals ethanol as the principal product, acetaldehyde as a persistent but smaller product, ultimately reflecting a shallow potential. Acetylene and ethylene appear as minor products only at −2.0 V. All products form with relatively similar FEs, and the ethylene-precursor is noticeable only at the most negative potential. This path is fed by *CH generation, hence becoming most important as *CH coverage grows at very negative potentials (Figure 7b).

**Figure 7:**
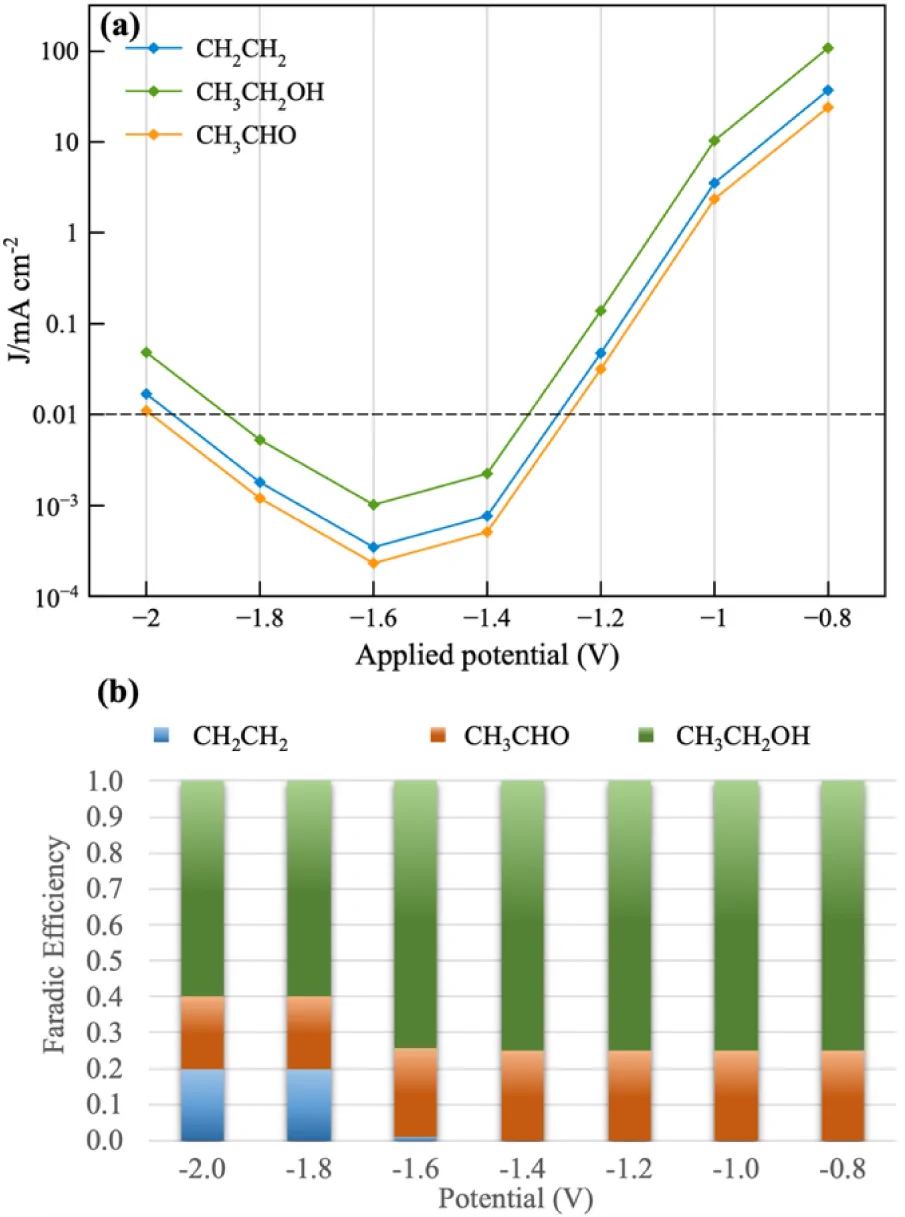
(a) Current density as a function of potential for C2 products, associated with the acetaldehyde path from −2.0 to −0.8 V. (b) Faradaic efficiencies within the acetaldehyde manifold. Here, ethylene formation is noticeable only at −2.0 V. Otherwise, ethanol remains present at 0.75–0.80 FE, and acetaldehyde holds from 0.20 to 0.25.

C2 product formation can be rationalized through two major points. First, absolute C_2_ currents around −1.2 V are relatively high (10^–2^–10^–1^ mA cm^–2^ for individual labeled species), where coupling reservoirs are most populated; currents increase by 1–3 orders of magnitude as potential moves to −1.0 or −0.8 V, consistent with an increased supply of coupled fragments.

In this regard, oxygen-rich liquids formed from the MOF are formed through the glyoxal pathway, which diverts steady-state coverage from the vinyl path. The vinyl path is the most direct path to ethanol, while the glyoxal path is governed by the formation of oxygen-rich liquid fuels formed after conversion from glyoxal to glycolaldehyde to ethylene glycol. Altering the bias associated with product formation, selectivity can move from ethylene-leaning to ethanol-leaning, then to oxygenate rich.

### Competition with Gas Products: Ethanol vs Ethylene

For the formation of ethanol and ethylene in the vinyl-alcohol manifold, the single rate-determining step for both is the formation of the *CCHOH intermediate (Figure 8a). Upstream steps that supply this basin are downhill and either barrierless or only weakly activated. Thus, the only one small energy barrier of 0.27 eV is for *CHCHOH → *CHCH_2_OH. The ethanol pathway proceeds by hydrogenating vinyl-alcohol into the β-hydroxy-ethyl intermediate and then to ethanol with no new kinetic barriers. Figure 8a displays this as well. The ethanol path is strongly exergonic and barrierless, with the network separating after formation of the *CCHOH intermediate. First, vinyl C–H addition *CCH → *CCH_2_ requires Ea = 0.48 eV and is endergonic (ΔG = +0.27 eV). Second, when the surface accommodates the acetylene path (which is available), *CCH → *CHCH is barrierless and exergonic (ΔG = −1.48 eV), while the subsequent hydrogenation *CHCH → *CHCH_2_ requires Ea = 0.34 eV (ΔG = −0.03 eV, nearly thermoneutral). Only after *CHCH_2_ is formed does the final step *CHCH_2_ → *CH_2_CH_2_ drop way downhill (ΔG = −6.75 eV, barrierless), reflecting the quenching of the open-shell vinyl fragment into closed-shell, weakly adsorbed ethylene (see Methods). Cross-path eliminations also siphon flux to the hydrocarbon branch from the ethanol branch, alongside the specialized ethylene steps presented here.

CHCHOH*→CHCH*isbarrierless(ΔG=−0.86eV)

CH2*CHOH→CHCH2*(ΔG=−2.78eV)barrierlesswatereliminationand

CHCH2*OH→CHCH2*(ΔG=−2.56eV)barrierlesswatereliminationsteps

**Figure 8:**
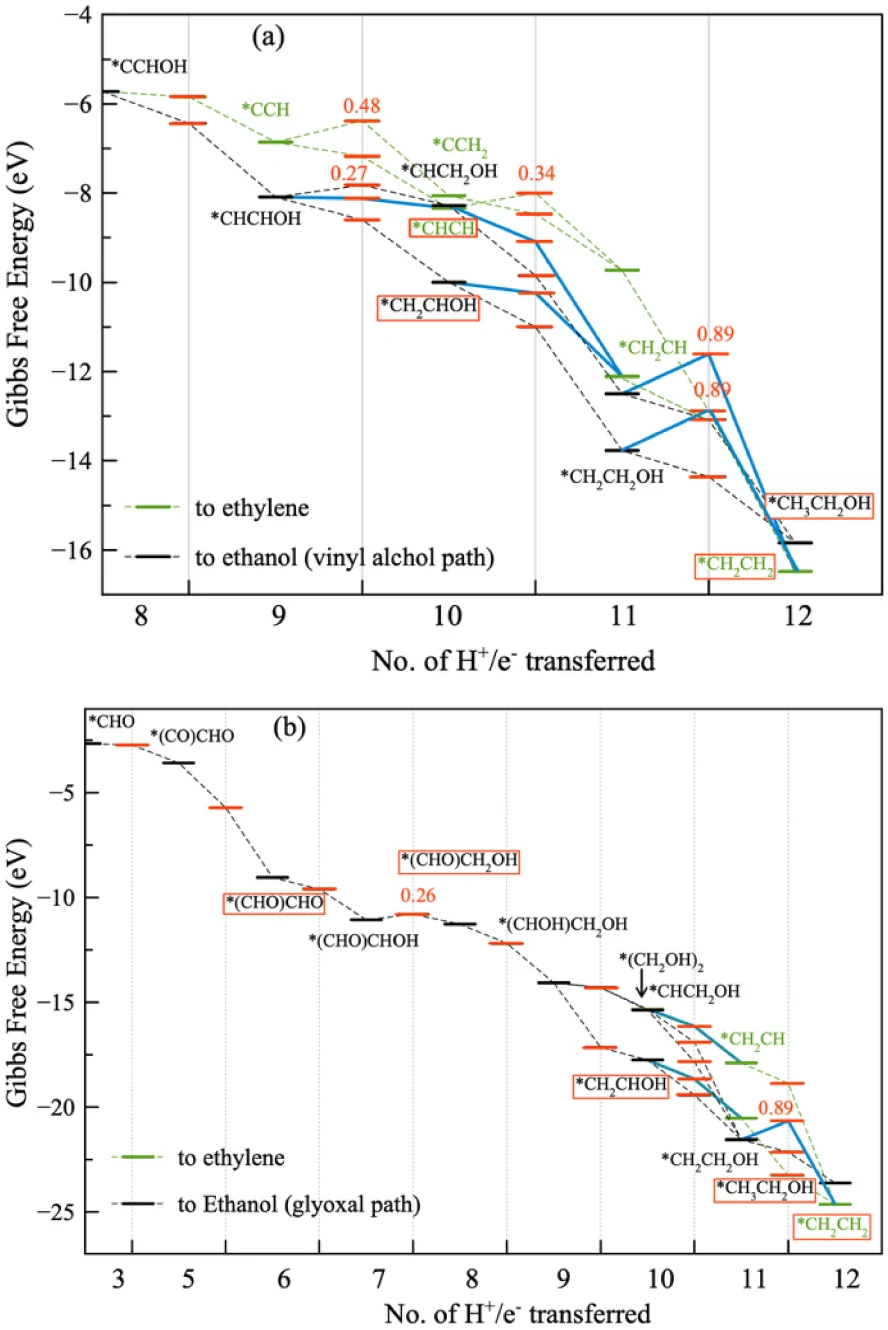
Grand-canonical free-energy diagram at −1.20 V contrasting the competition between the (a) vinyl alcohol and (b) glyoxal pathways in the formation of ethanol and ethylene. Green dashed lines indicate the competing surface dehydrogenation pathway to ethylene, while black dashed lines indicate the formation of ethanol. Adsorbed intermediates are marked at each vertex, and the total number of transferred proton–electron pairs serves as an index of reaction progress. Forward activation barriers are indicated by horizontal red bars (values in eV printed above). The water elimination stages where the ethanol and ethylene paths intersect are indicated by the thick cyan lines.

But when we consider the β-hydroxy-ethyl step, the “last-chance” dehydration to ethylene is very unfavorable: *CH_2_CH_2_OH → *CH_2_CH_2_ with the barrier Ea = 0.89 eV (ΔG = −2.31 eV). While the thermodynamic driving force for ethylene is larger than for ethanol, we see that the kinetic profile from the common intermediate favors ethanol over ethylene. There are two barriers of 0.48 and 0.34 eV for formation of ethylene, but only continued barrierless steps for the formation of ethanol. Thus, the yield for ethylene comes from the barrierless cross-path eliminations before the surface dips irreversibly into the β-hydroxy-ethyl/ethanol basin, as shown in Figure 8b.

After dimer formation and initial protonation to *­(CHO)­CHOH, the key step is the second carbon-side protonation to form *­(CHO)­CH_2_OH with a small Ea = 0.27 eV and only a slight downhill driving force (ΔG = −0.21 eV), which explains the accumulation of glycolaldehyde in FE. From there, two barrierless pathways are available; the first consisting of protonation of the oxygen to the ethylene-glycol precursors, and the second consisting of dehydration back onto the vinyl path via *CHCH_2_OH (or *CH_2_CHOH) → *CH_2_CH_2_OH. For the vinyl alcohol path, ethylene can still appear through the same cross-path eliminations, which are barrierless. Once CH_2_CH_2_OH is formed, the high 0.89 eV dehydration barrier again suppresses conversion to ethylene. Minor cross barriers are present in the final acetaldehydes path, present only in the vinyl alcohol branch (see Figure S2 in SI).

### Competition between C1 and C2 Products

Competition between C1 and C2 products is governed by two coupled factors, the first of which involves the entry to *CHO, and the second involves the availability and amount of the CO/CHO reservoirs that lead to C2 branches in the network. Across the potential window, formation of *HOCO is the bottleneck, followed by barrierless *CO and facile *CHO formation. From *CHO, C1 flux accelerates through a largely barrier-light cascade to methanol whereas methane is throttled by formation of *CHOH from *CHO. C2 formation occurs once *CO/*CHO coverage rises enough to stabilize the dimeric ketenyl core *­(CHO)­CO. Two topologies dominate. On the vinyl-alcohol map, all steps via the common node *CCHOH are downhill and barrierless or weakly activated. From *CCHOH the network diverges: the ethanol branch proceeds via barrierless hydrogenations, but the ethylene branch presents two high activation barriers, as described above. For the glyoxal pathway, the only relevant energy barrier is the *­(CHO)­CH_2_OH formation, after which reconvergence to the vinyl/β-hydroxy-ethyl basin is barrier-free. These energy distinctions are directly reflected in potential-dependent partial currents: C2 marker currents have a deep near −1.6 V in the range 10^–3^–10^–2^ mA cm^–2^, then increase by 1–3 orders of magnitude toward −1.2 and −0.8 V as the coupled reservoir is depleted. At the same potentials, the methanol cascade in C1 remains rapid (post-*CO barriers ≤ 0.27 eV) and thus competes away dimerization unless *CO/*CHO coverages are high.

These free-energy plots show that for C1 the sequence after *CHO formation has just the 0.27 eV barrier to methanol, whereas the methane path carries the 0.50 eV *CHOH barrier penalty. Thus, at moderate bias, *CHO is converted more rapidly into methanol than it is reduced to methane or built up into dimers. For C2, the vinyl channel shows reversed asymmetry after the bifurcation: the ethanol route remains barrier-light (no further positive spans), while the ethylene route must overcome 0.48 and 0.34 eV barriers. The glyoxal path adds a small activation energy (0.26 eV) for glycolaldehyde/glyoxal formation but continues toward barrierless ethanol formation. A final dynamic feature that quenches “late” ethylene is the huge dehydration barrier from *CH_2_CH_2_OH (0.89 eV). Despite these penalties, ethylene is observed experimentally because a set of barrierless cross-path eliminations siphon off flux from ethanol-bound intermediates before the β-hydroxy-ethyl formation. These channels are responsible for the persistent, though secondary, ethylene current in the C2-resolved J–V plots. They also connect the manifolds: fast C1 hydrogenations feed CH/CHO pools that can couple, but the same fast hydrogenations also drain the vinyl pool before ethylene’s 0.34–0.48 eV activation energies are overcome, resulting in C1 and C2 product selectivity differences.

## Conclusions

The grand-canonical microkinetic analysis presented here offers a quantitative, atomistic view of how phthalocyanine-based 2D-MOFs steer the electro-reduction of CO_2_ toward either gaseous or liquid solar-fuel products. We validated the behavior of our catalyst through a stiff ODE solver, exposing product selectivity under a variety of different potential biases. As opposed to thermodynamics and the presence of thermodynamic driving product formation, we find that kinetic bottlenecks dictate the behavior of the 2D-MOF in question, with methanol outcompeting methane at moderate overpotentials. A similar behavior appears in the C2 network, where the ketenyl route to ethanol provides a barrier-less C–C coupling channel, while ethylene initially profits from a facile dehydrogenation but eventually yields to ethanol once the hydrogenation barrier for the rate-controlling *­(CHO)­CH_2_OH formation step of the glyoxal branch collapses from 0.26 eV at −1.20 V to 0.15 eV at −1.80 V. These mechanistic insights, obtained directly from the grand-canonical method, rationalize the experimentally observed shift from C1 liquids to C2 gases and, at more negative potentials, to C2 liquids. We see that the decisive intermediates in each case are the *CHO and *CCHOH, whose formation rise faster at more negative potentials. The presence of *CHO and *CCHOH intermediates unlocks the ketenyl and acetyl pathways that lead into C2 products. As a result, the catalyst responds in a variety of different fashions when exposed to different input biases:

- the potential window from −1.2 to −1.5 V leads to methanol product domination,
- the window from −1.5 V to −1.75 V leads to ethanol superseding both C1 liquid products and methane,
- beyond −1.8 V ethylene begins to develop in competition with ethanol.

Building on the potential-dependent BEP formalism developed by Akhade and Janik,^26^ we couple BEP-derived barriers with grand-canonical free energies obtained from JDFTx/CANDLE to construct a microkinetic network spanning more than 50 elementary steps on a single-atom 2D-MOF catalyst. The resulting kinetic matrices are exported to an open microkinetic workbook as a reproducible blueprint for future catalyst investigation and discovery. We anticipate that the work presented here will accelerate the rational design of next-generation MOF catalysts, allowing for eventual practical deployment for sustainable fuel synthesis in industrially relevant conditions for the widespread adoption of MOFs in the near future.

In regards to future work, experimental analyses can be utilized to better understand the relationship between the kinetics, potential bias, and product selectivity; identifying the existence between distinct kinetic “windows” to which specific products are formed, and intermediates. Specifically, operando vibrational spectroscopy, notably surface-enhanced infrared absorption spectroscopy (SEIRAS) and Fourier Transform Infrared Spectroscopy (FTIR) can be used to confirm the emergence of various bonds either broken or formed at the surface of the catalytic MOF, to confirm or denying whether specific crucial intermediates have been formed. From FTIR, characteristic bands associated with intermediate product bond stretching and deformation modes can also be used to confirm the stabilized intermediates that lead to final product formation, with operando RAMAN/SERS spectroscopy supporting such intermediates as well by confirming whether specific C–O/C–C containing intermediates exist through vibrational fingerprints, in addition to using isotopic labeling to confirm carbon provenance. These experiments should confirm which intermediate products are formed in the creation of final liquid solar fuels. For final product formation, differential electrochemical mass spectrometry (DEMS) can be used to produce high-resolution faradaic efficiency maps for C1 and C2 products, testing whether product formation occurs over the potential windows specified. FE mapping and kinetic diagnostics in the form of Tafel mapping can be used to model whether the microkinetic-structure developed is consistent with experimental analyses, with the windows predicted leading to the products specified. These experiments, that can verify the theory predictions, are the next steps from the work presented here.

## Supplementary Material



## Data Availability

Figures and tables including parameters for the GCP-K method, reaction pathways at acid pH, additional electronic properties and microkinetic analyses can be found within the Supporting Information. Correspondence and requests for materials should be addressed to s.osella@cent.uw.edu.pl

## Notes

Schematic details of the system used in the study; reaction pathways for the different competing processes; potential-dependent driving forces for the rate-controlling proton-coupled electron-transfer (PCET) steps in the CO2 RR microkinetic network; bond distances, GCP, and microkinetic parameters for all the studied intermediates (PDF)
