# Supplementary material for: Competition between Gas and Liquid Solar Fuel Product Formation from the CO2 Reduction Reaction on Two-Dimensional Metal-Organic Framework
Source: J Phys Chem C Nanomater Interfaces. 2026 Aug 6;130(33):11634–44. doi: 10.1021/acs.jpcc.6c03863 (PMC13501952; doi:10.1021/acs.jpcc.6c03863)
Supplement: Supplementary file 1 [file jp6c03863_si_001.pdf]

Suplementary information for

Competition Between Gas and Liquid Solar Fuel Product Formation from the CO_2 Reduction Reaction on Two-Dimensional Metal Organic Frameworks

Aarya D. Riasati,[2] William A. Goddard III,[2] Silvio Osella*[1,2]

[1] Materials and Process Simulation Center (MSC), California Institute of Technology, MC 139-74, Pasadena CA, 91125, USA.

[2] Chemical and Biological Systems Simulation Lab, Centre of New Technologies, University of Warsaw, Banacha 2C, 02-097 Warsaw, Poland.

Email: s.osella@cent.uw.edu.pl

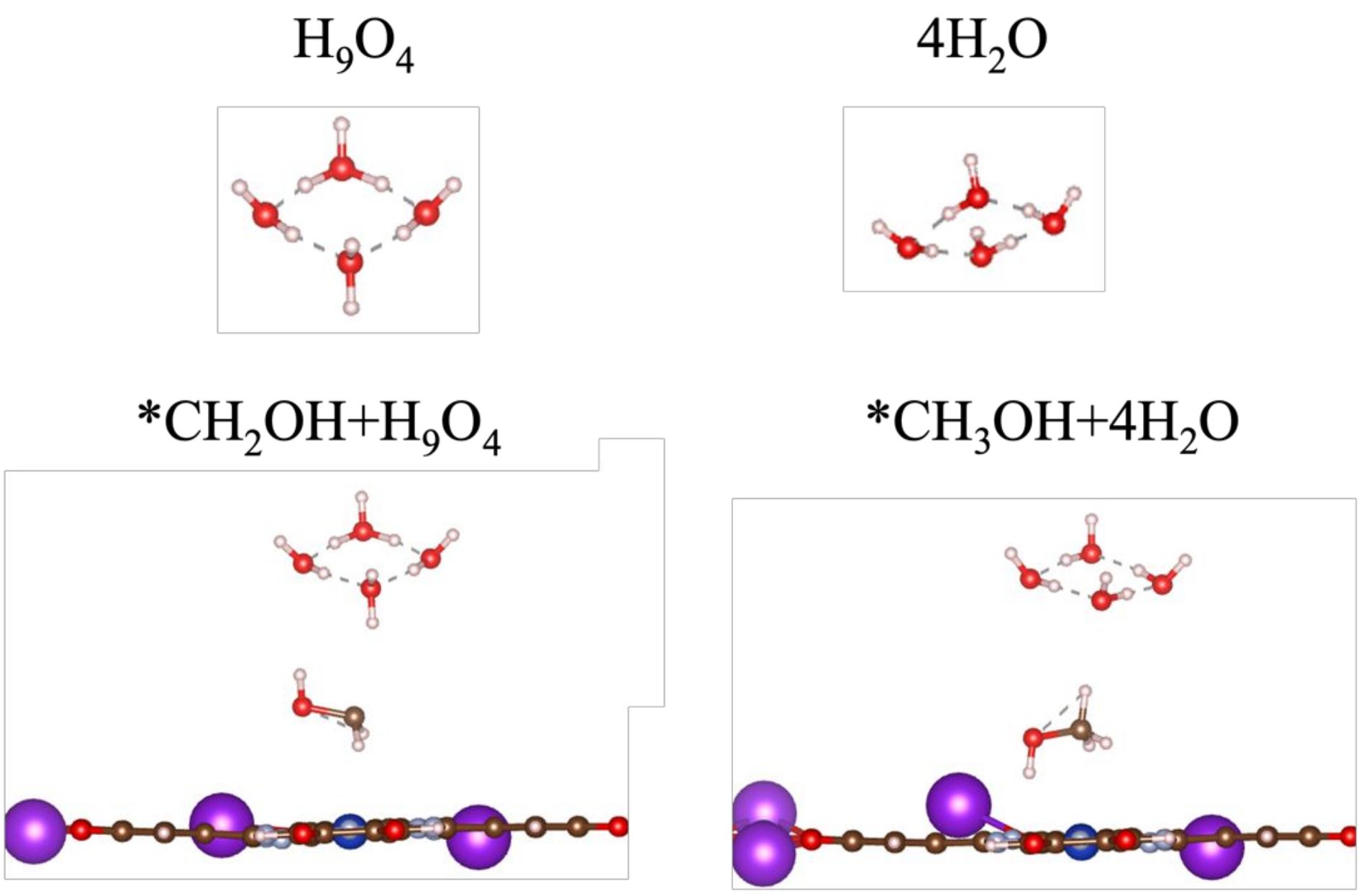

Figure S1. (*top*) Water cluster used for the calculations. For the reactant, the H_9O_4 cluster is consdiered, while for the product -after proton transfer- $4H_2O$ are present. (*bottom*) representative structure of the interaction of the water cluster with the adsorbate for the CH_2OH protonation to methanol.

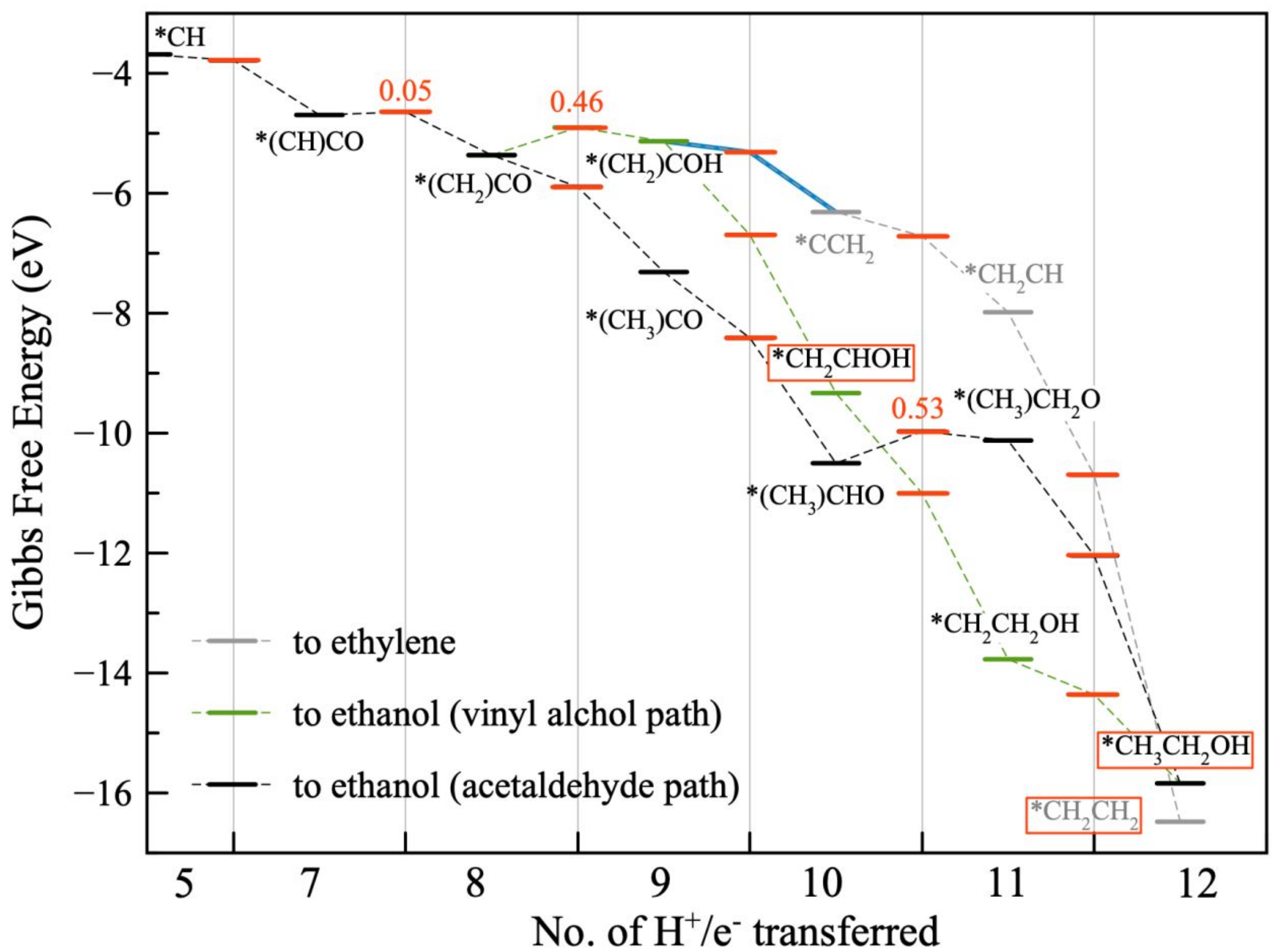

Figure S2. Grand-canonical free-energy diagram at -1.20 V versus SHE contrasting the competition between the (a) vinyl alcohol and (b) acetaldehyde pathways in the formation of ethanol and ethylene.

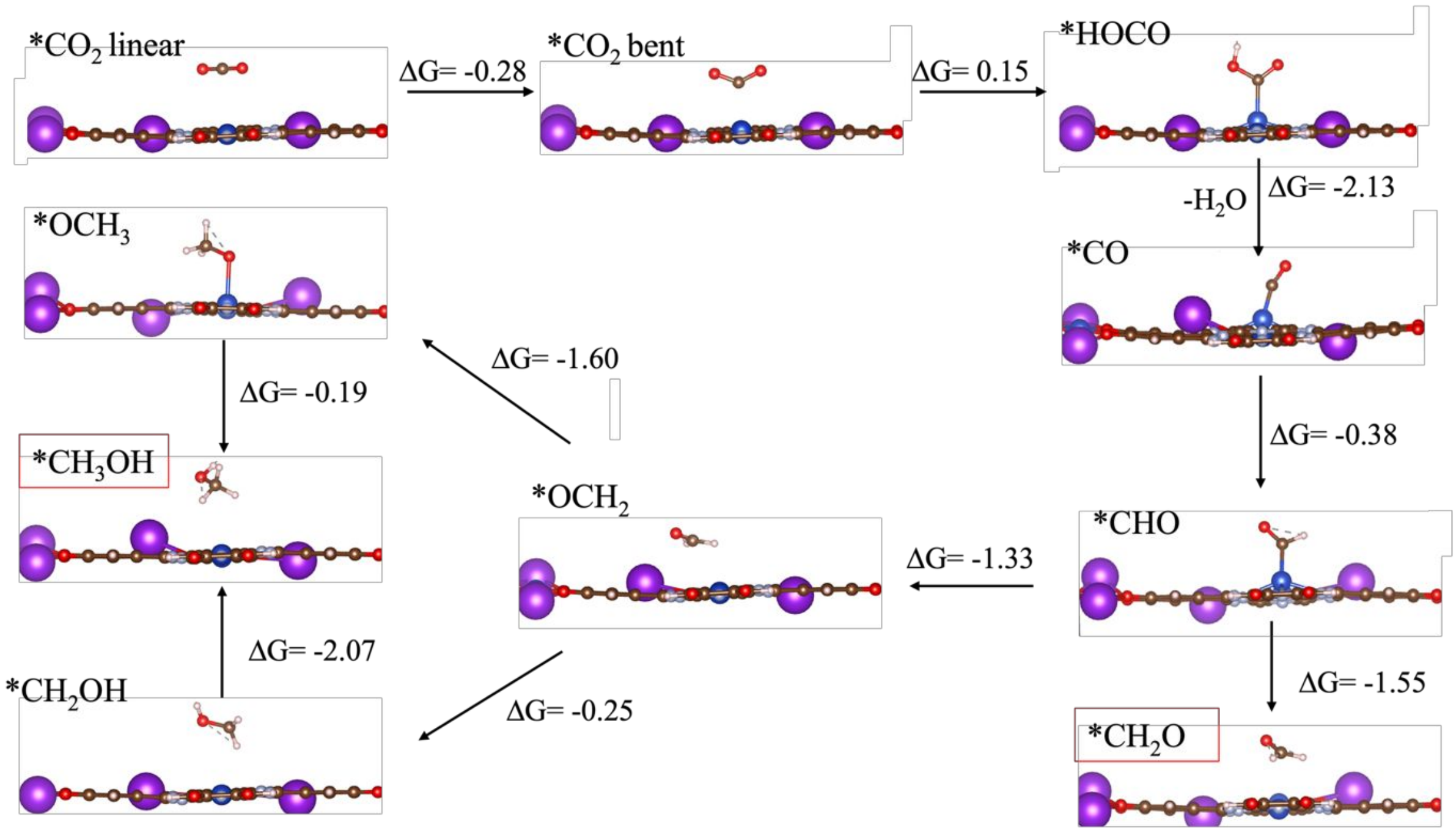

Figure S3. Representative structures of the intermediates for methanol formation. Gibbs free energy changes are reported for U=-1.2 V and pH=7, at 298 K (experimental conditions). All energies are in eV.

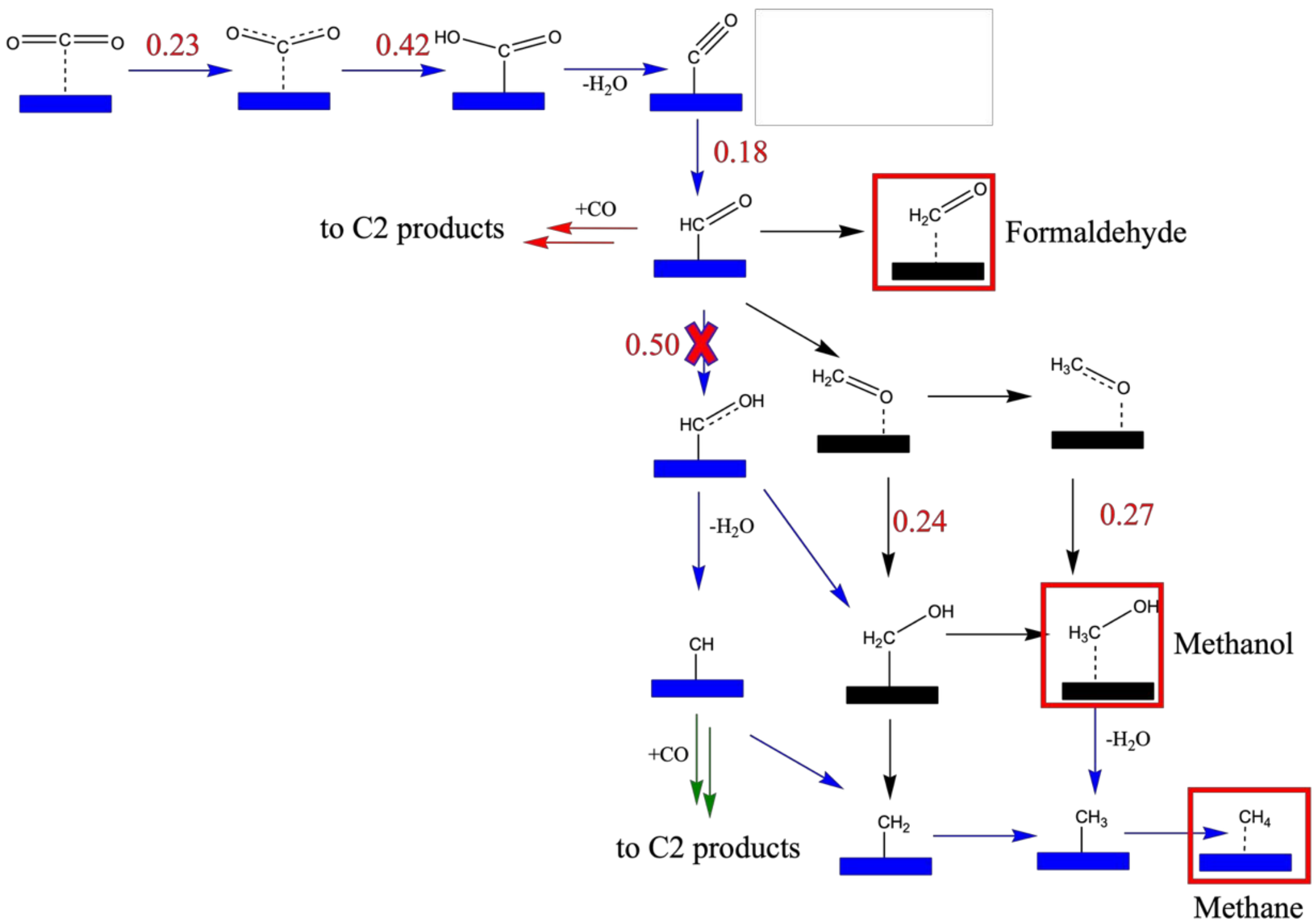

Figure S4. Annotated kinetic decision map for C1 product formation on the 2D-MOF surface at −1.20 V vs SHE. Line drawings depict adsorbed intermediates; the small plinth under each structure represents the slab. Red numerals give forward activation barriers in eV (from the grand-canonical BEP model). The rate determining step for the formation of both methane and methanol is the *HOCO protonation ($\Delta G^{\ddagger}$ = 0.42 eV). Black arrows and platforms trace the

downhill hydrogenation sequence that culminates in methanol, with a modest barrier of 0.27 eV. Blue arrows and platforms chart the competing dehydration route that yields gaseous methane; its rate-limiting step (*CHO → *CHOH) carries a larger 0.50 eV barrier. Red boxes mark key kinetic checkpoints, while red X symbols flag the branches that are kinetically disfavoured under the chosen bias (hihger than the initial RDS of 0.42 eV). Collectively, the diagram visualises how modest differences in barrier heights and the berrierless dehydration on the methanol path tip the C1 selectivity balance between methanol (blue) and methane (black).

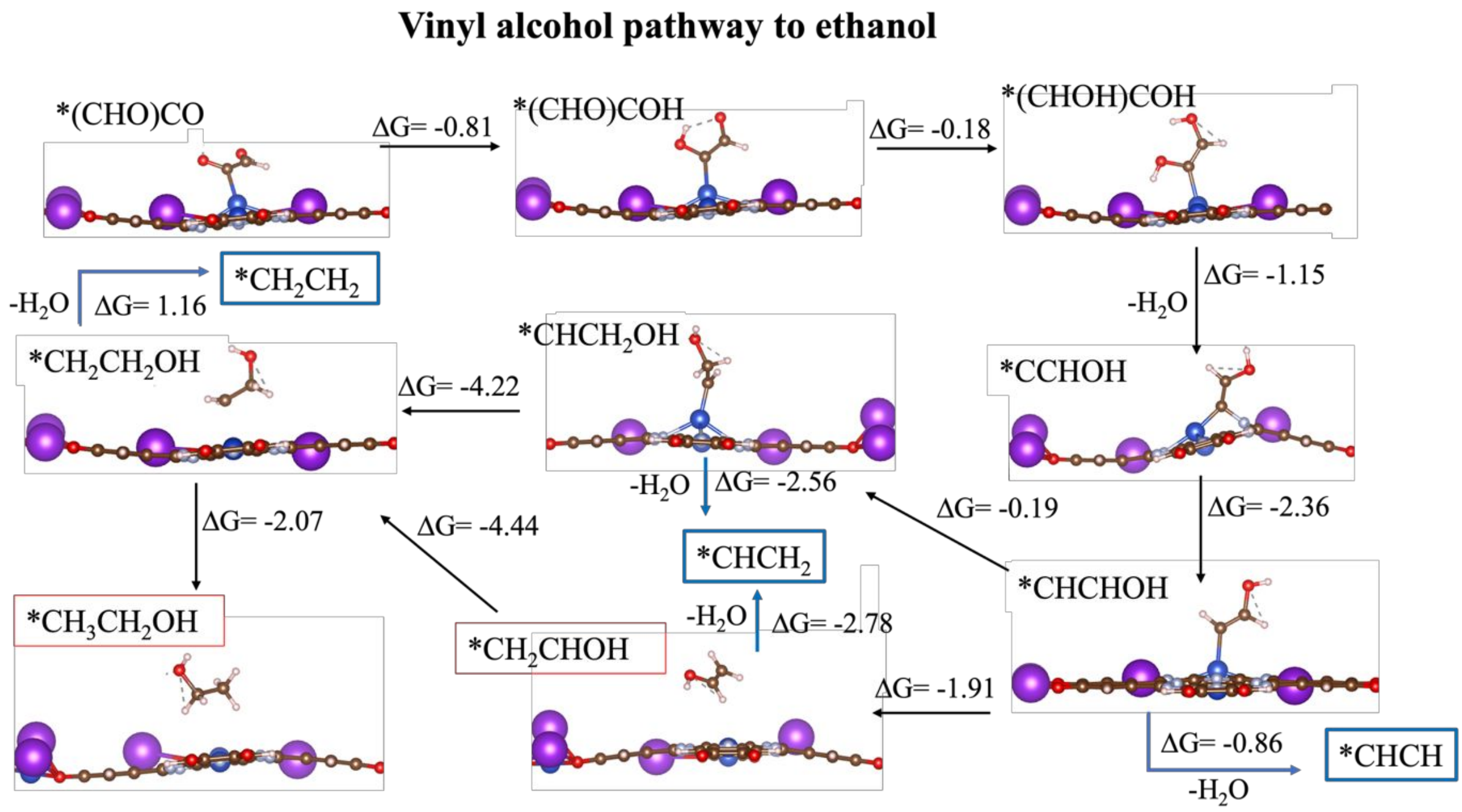

Figure S5. Representative structures of the intermediates for ethanol formation through the vinyl pathway. Gibbs free energy changes are reported for U=-1.2 V and pH=7, at 298 K (experimental conditions). All energies are in eV.

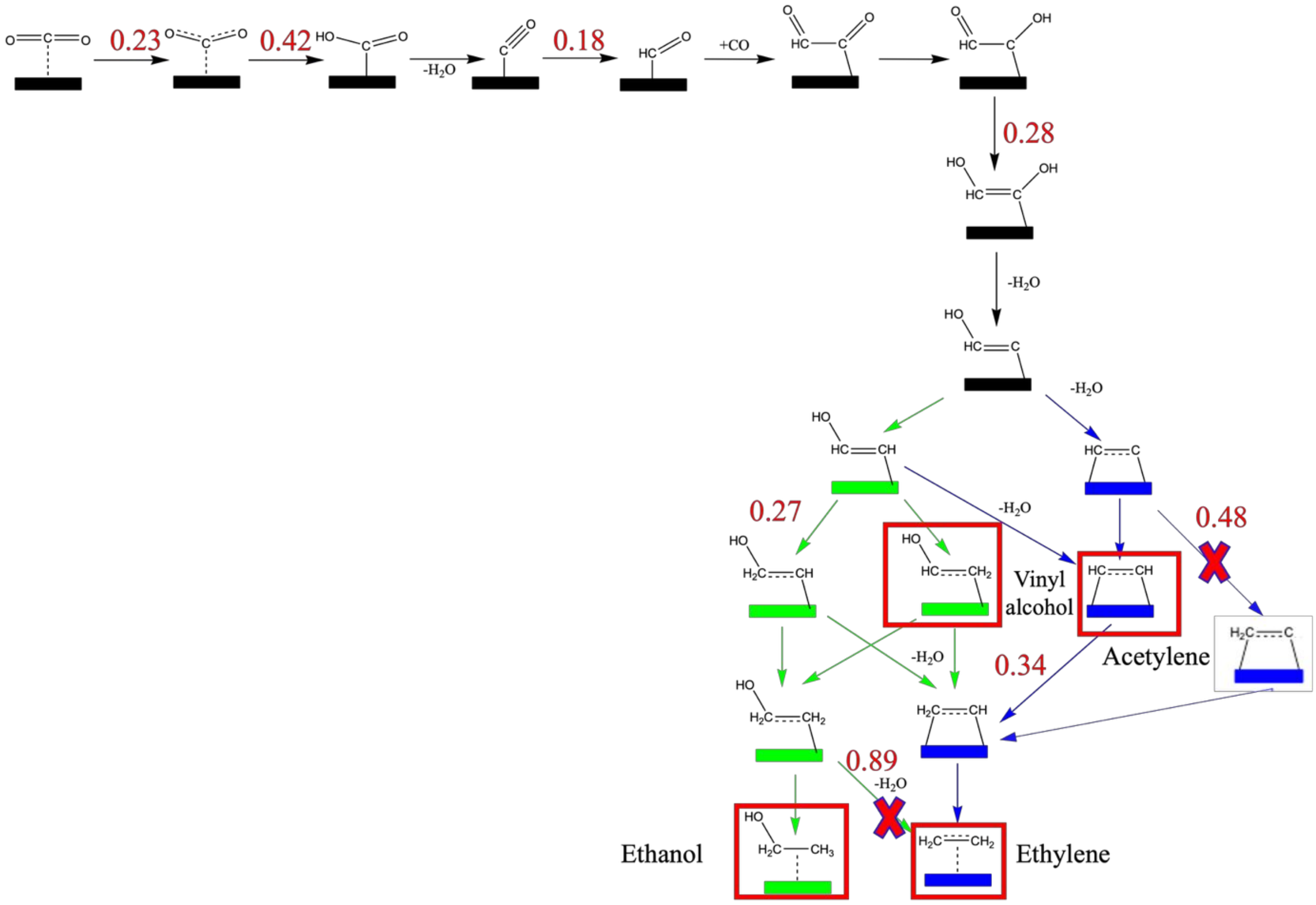

Figure S6. Annotated kinetic decision map for C2 product formation along the vinyl alcohol pathway at −1.20 V vs SHE. Line drawings depict adsorbed intermediates; the small black plinth under each structure represents the slab. Red numerals give forward activation barriers in eV (from the grand-canonical BEP model). Green arrows and platforms trace the downhill hydrogenation sequence that culminates in liquid ethanol; the highest barrier on this trajectory is the vinyl-to-allyl protonation ($\Delta G^{\ddagger}$ = 0.27 eV). Blue arrows and platforms chart the competing de-hydrogenation route that yields gaseous ethylene via vinyl alcohol; its rate-limiting protonation ($*CHCH \rightarrow *CHCH_2$) carries a larger 0.34 eV barrier. Red boxes mark key kinetic checkpoints, while red X symbols flag the branches that are kinetically disfavoured under the chosen bias (hihger than the initial RDS of 0.42 eV). Collectively, the diagram visualises how modest differences in barrier heights tip the C2 selectivity balance between ethylene (blue) and ethanol (green).

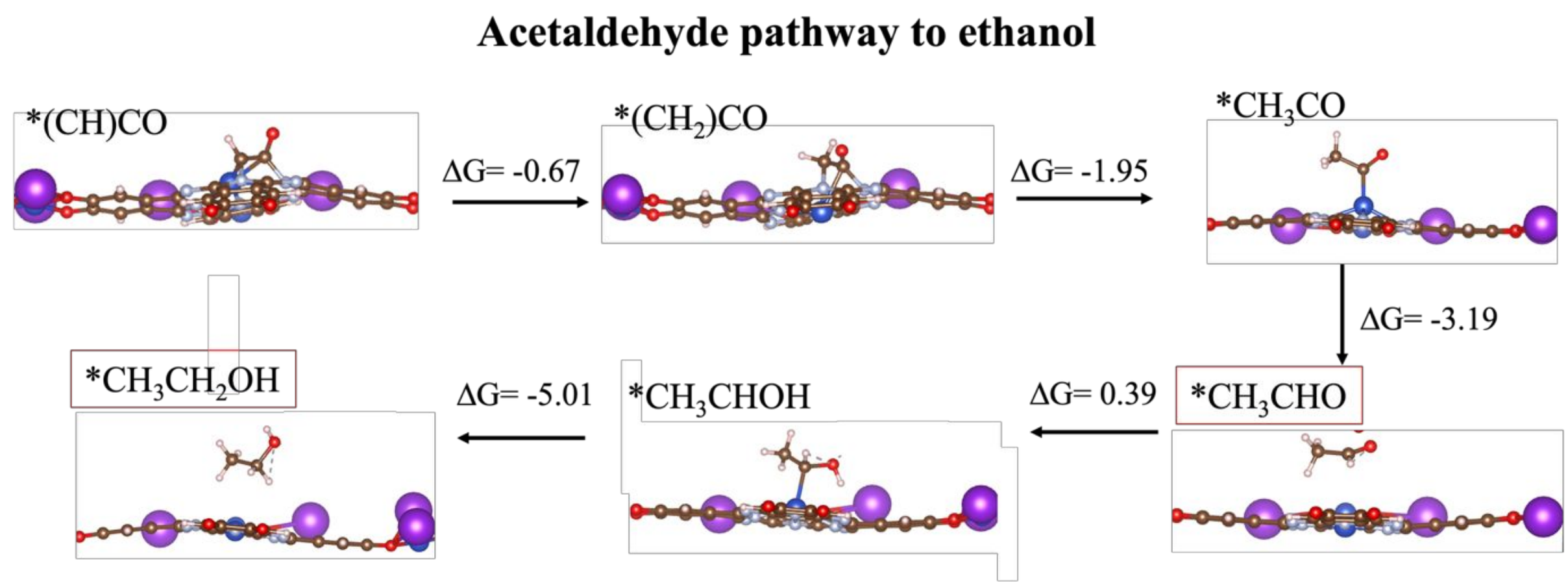

Figure S7. Representative structures of the intermediates for ethanol formation through the acetaldehyde pathway. Gibbs free energy changes are reported for U=-1.2 V and pH=7, at 298 K (experimental conditions). All energies are in eV.

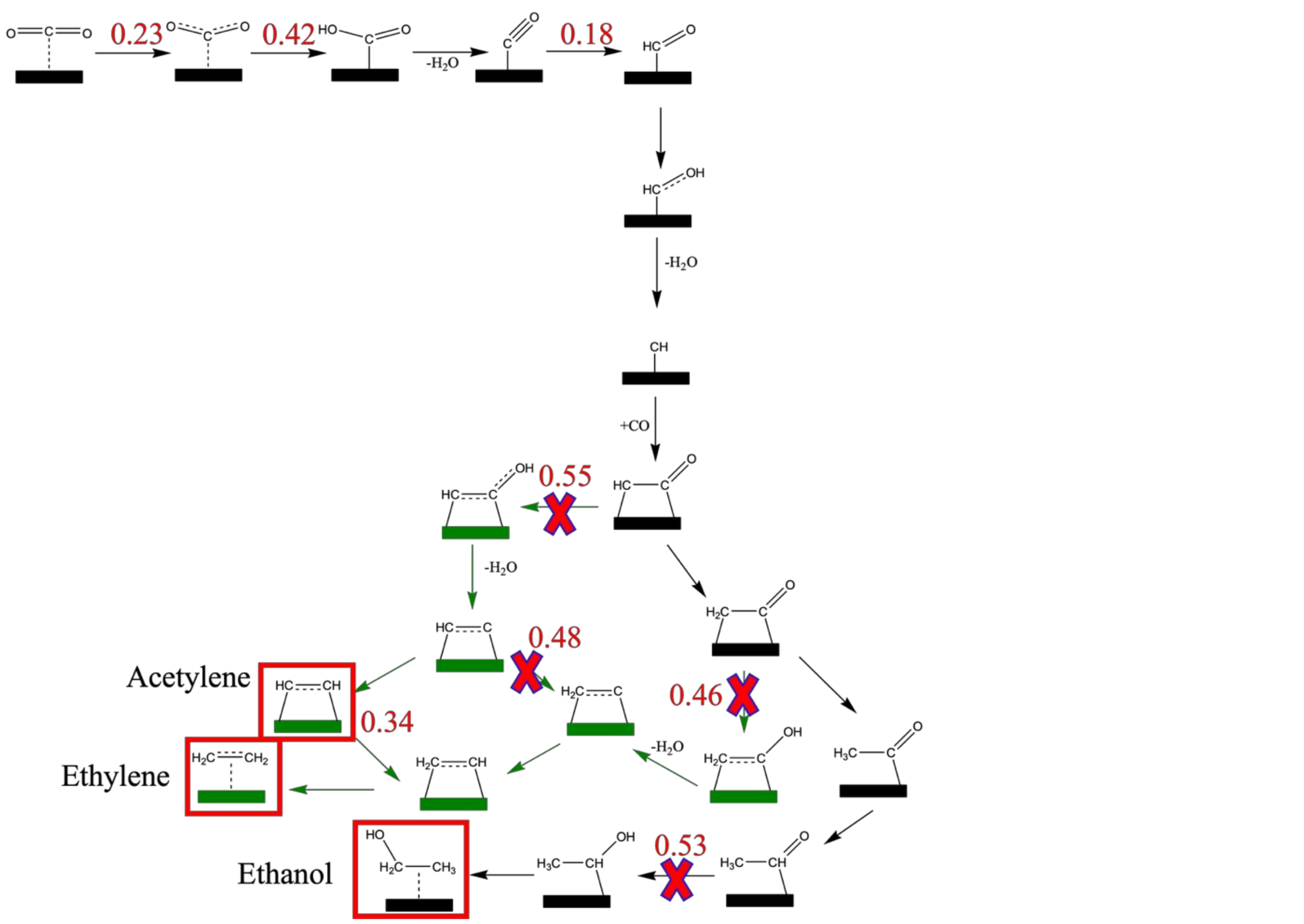

Figure S8. Annotated kinetic decision map for the *acetaldehyde* coupling manifold at −1.20 V vs SHE. Line drawings depict adsorbed intermediates; the small black plinth under each structure represents the slab. Red numerals give forward activation barriers in eV (from the grand-canonical BEP model). Green arrows and platforms trace the downhill hydrogenation sequence that culminates in ethylene. Black arrows and platforms chart the competing liquid ehtanol via vinyl alcohol. Red boxes mark key kinetic checkpoints, while red X symbols flag the branches that are kinetically disfavoured under the chosen bias (hihger than the initial RDS of 0.42 eV).

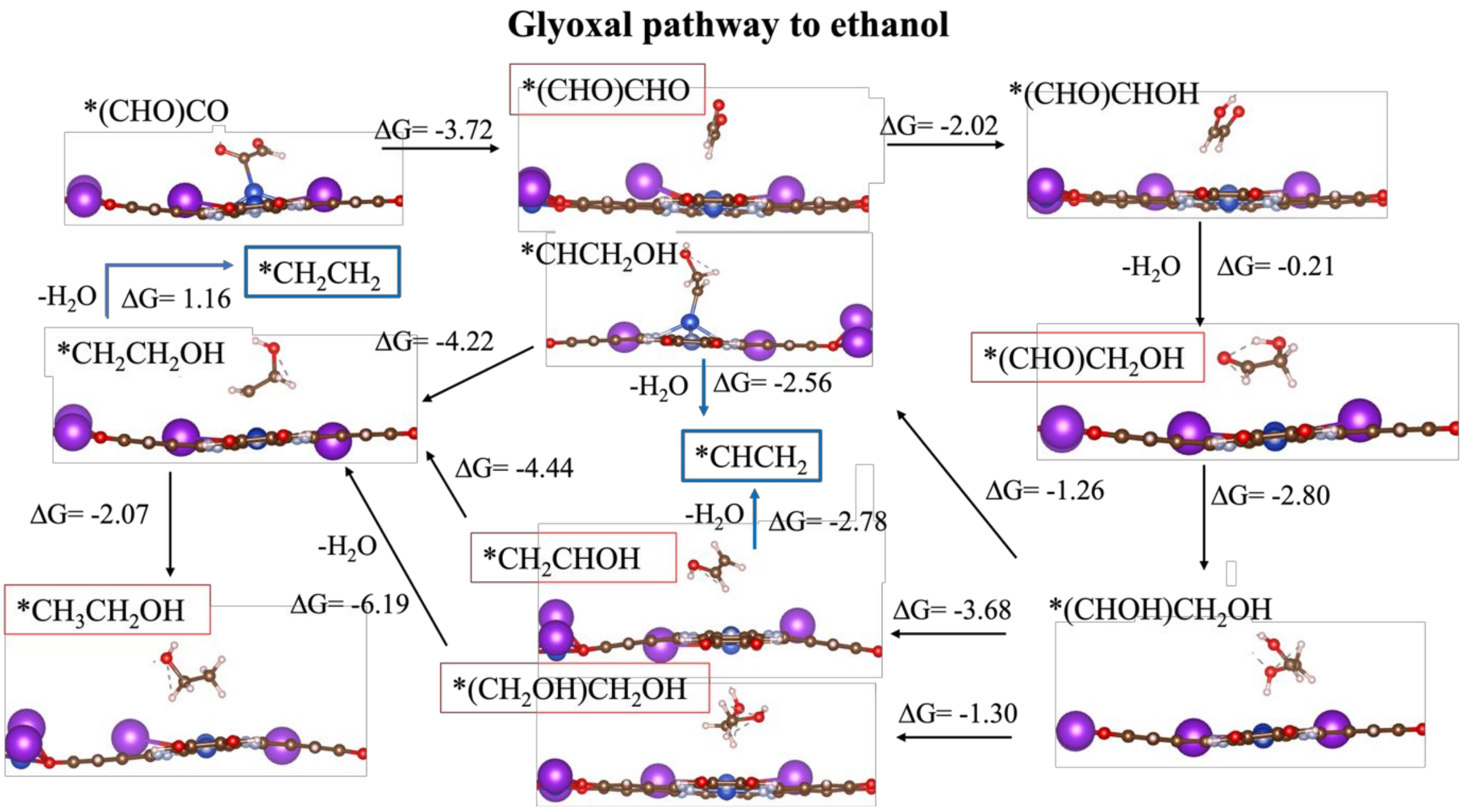

Figure S9. Representative structures of the intermediates for ethanol formation through the glyoxal pathway. Gibbs free energy changes are reported for U=-1.2 V and pH=7, at 298 K (experimental conditions). All energies are in eV.

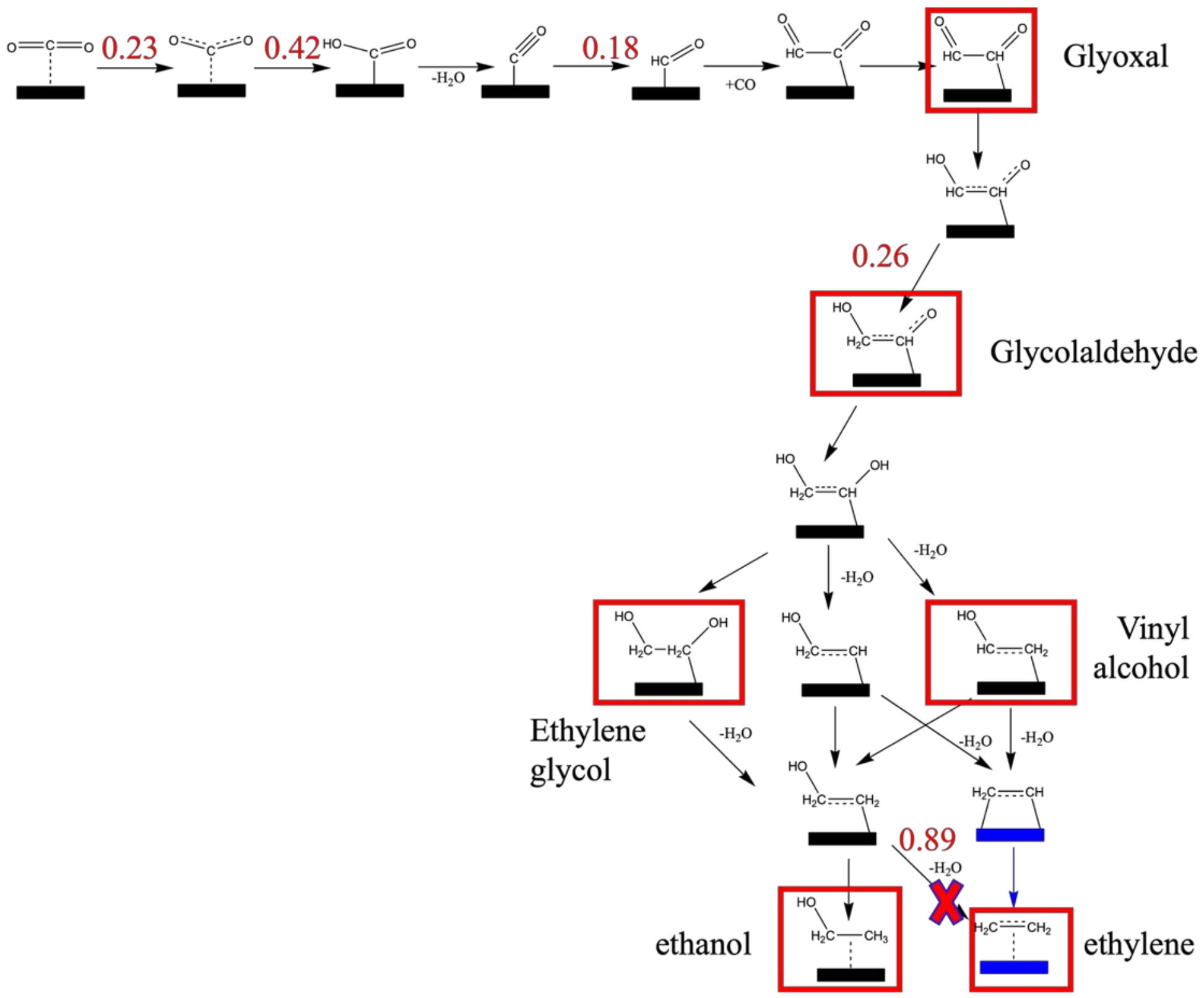

Figure S10. Annotated kinetic decision map for the *glyoxal* coupling manifold at –1.20 V vs SHE. Stick sketches depict adsorbed intermediates resting on the 2D-MOF slab (black plinth). Red numerals are forward activation barriers in eV extracted from the grand-canonical BEP model. Red boxes enclose the principal kinetic checkpoints, glyoxal, glycolaldehyde, ethylene glycol, vinyl alcohol and the terminal ethanol precursor, while blue arrows trace the competing

dehydration route that yields gaseous ethylene. The sequence begins with three modest barriers that generate glyoxal; a single 0.26 eV hydrogenation converts it to glycolaldehyde, after which parallel channels diverge: successive hydrogenations (black arrows) proceed smoothly toward ethylene glycol and ultimately to surface-bound ethanol, whereas serial dehydrations (blue) encounter a steep 0.89 eV hurdle en route to ethylene. Red X marks the kinetically disfavoured branch at this potential. The diagram visualises how the relatively low 0.26 eV barrier tips this pathway toward liquid ethanol while throttling ethylene production under moderate overpotentials.

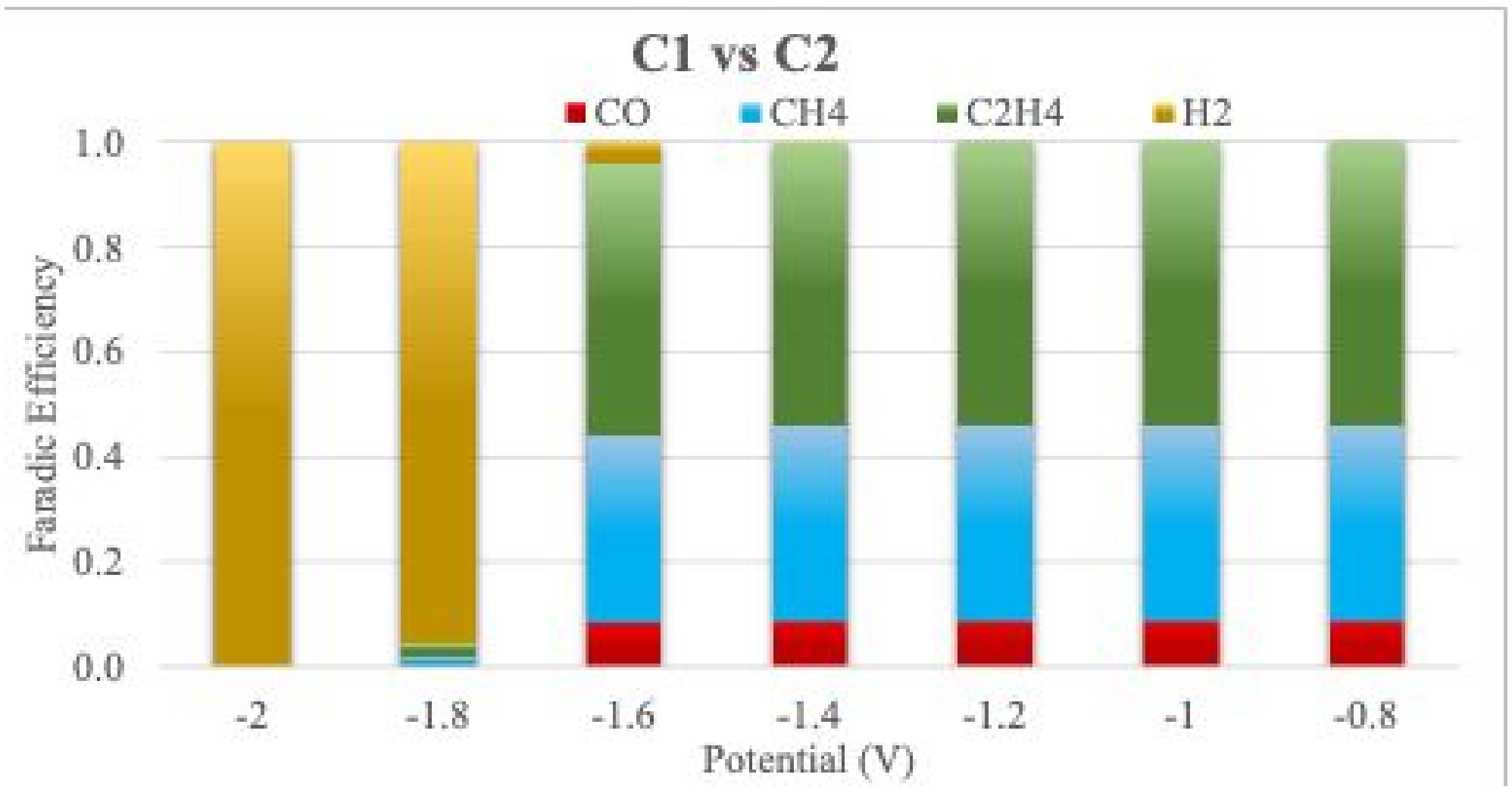

Figure S11. Faradic efficiency comparison between C1 and C2 gaseous products. Comparing our results with the experimentally available data, reported in [JACS 2021, 143, 19, 7242–7246], we can see that our computational methodology reproduces the ethylene formation of 54% at -1.2 V, and the 37% formation of methane. While the ehylene production is in exellent agreement with the experiment (reported as 50%), we overhestimate the methane formation, due to the lack of hydrogen production at low biases. In contrast, the experimental data show a 30% H_2 production at the applied bias of -1.2V. This discrepance is due to the fact that the reported data for HER are preliminary.

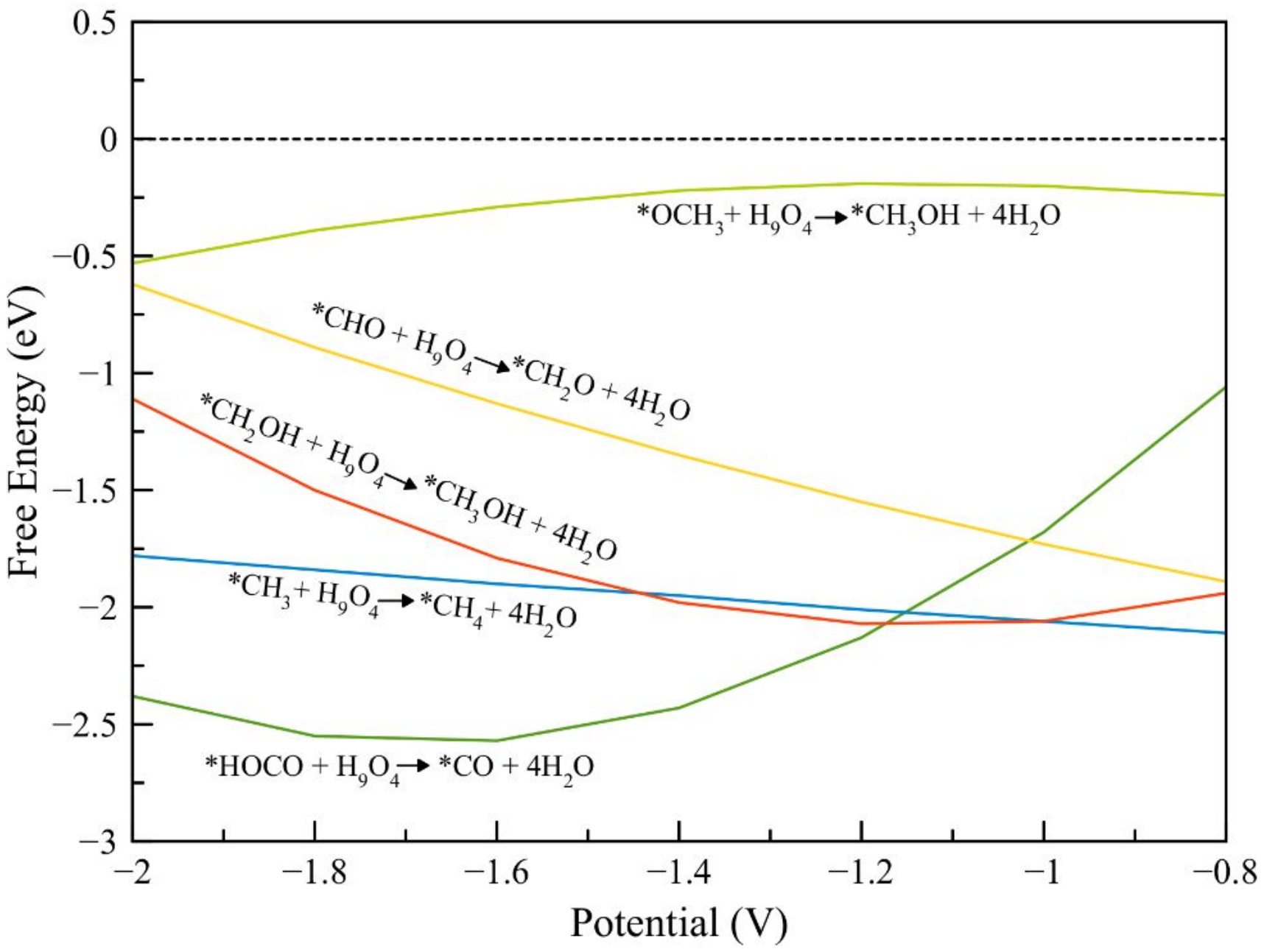
*OCH3+ H9O4 → *CH3OH + 4H2O
*CHO + H9O4 → *CH2O + 4H2O
*CH2OH + H9O4 → *CH3OH + 4H2O
*CH3+ H9O4 → *CH4+ 4H2O
*HOCO + H9O4 → *CO + 4H2O
Free Energy (eV)
Potential (V)

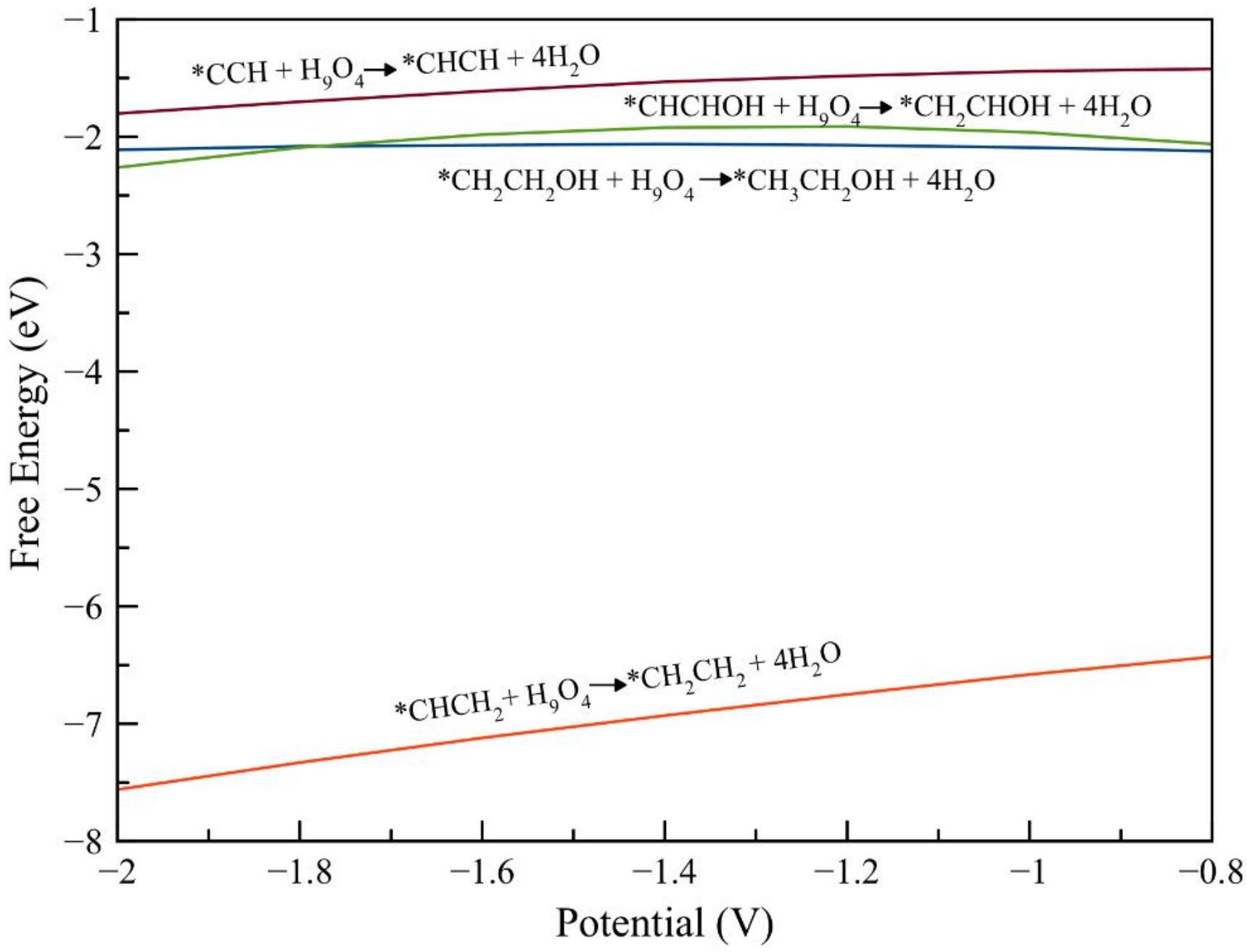
*CCH + H9O4 → *CHCH + 4H2O
*CHCHOH + H9O4 → *CH2CHOH + 4H2O
*CH2CH2OH + H9O4 → *CH3CH2OH + 4H2O
*CHCH2+ H9O4 → *CH2CH2 + 4H2O
Free Energy (eV)
Potential (V)

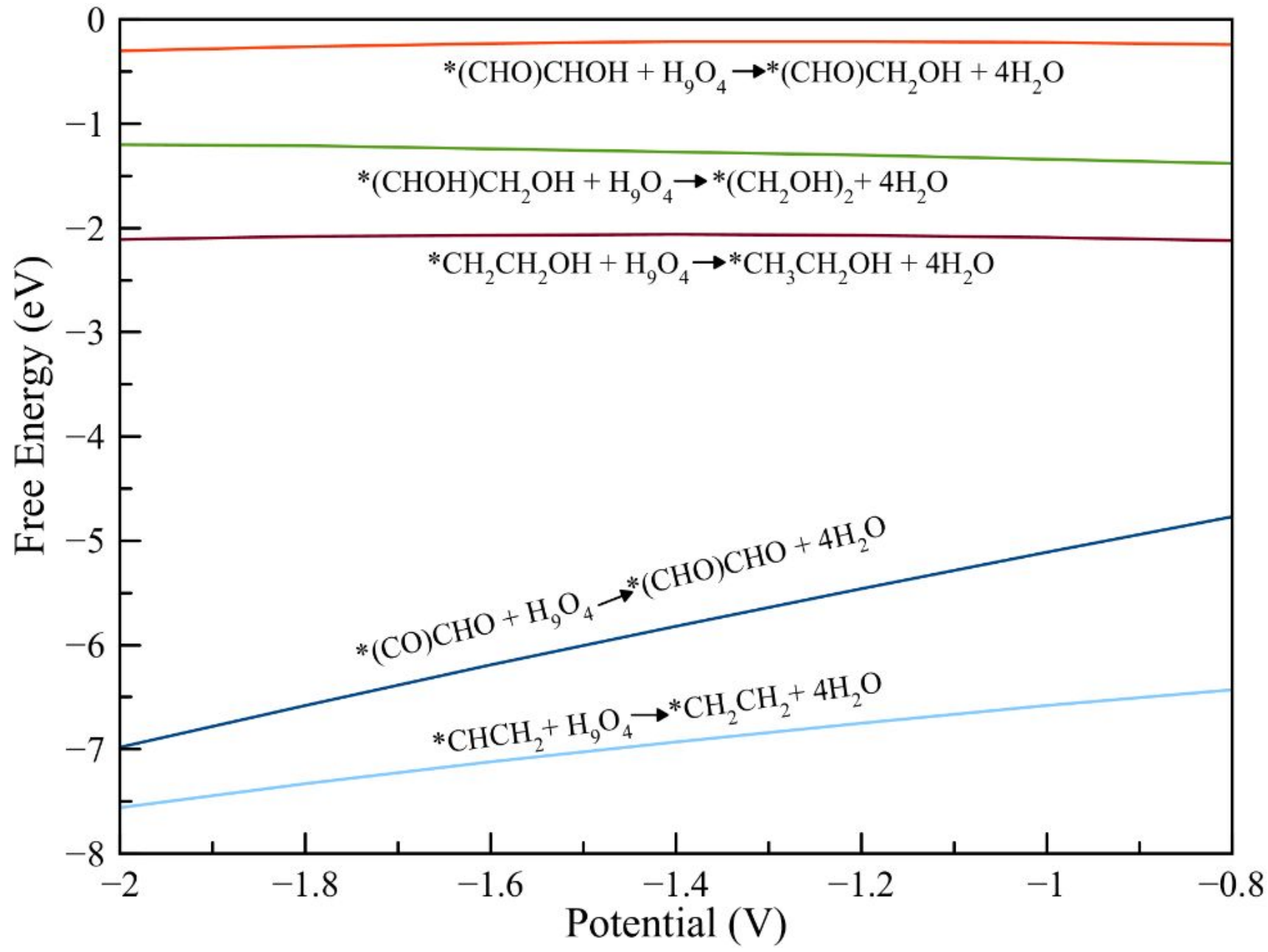

Figure S12. Potential–dependent driving forces for the rate-controlling proton-coupled electron-transfer (PCET) steps in the CO2 RR micro-kinetic network.
(a) C1 branch. Free-energy profiles for the five decisive hydrogenations that govern flux to methanol (green and orange traces) and methane (red and blue traces), plus the earliest *HOCO → *CO reduction (dark-green). All reactions become increasingly exergonic with cathodic bias, but to very different extents: the *CHO → $*CH_2O$ and $*CH_2OH$ → $*CH_3OH$ steps gain ≈1 eV between −0.8 and −1.8 V, whereas the final $*OCH_3$ → CH_3OH hydrogenation remains weakly uphill even at −2.0 V, flagging it as the residual throttle on methanol release. In contrast, the $*CH_3$ → CH_4 transformation is already downhill by −2.1 eV at −1.2 V, consistent with methane's facile desorption once the upstream *CHOH barrier has been overcome.
(b) Vinyl alcohol C2 branch. Bias sensitivity of the four key PCETs that interconvert vinyl (*CHCH), β-hydroxy-vinyl ($*CH_2CHOH$), ethylene ($*CH_2CH_2$) and the ethanol precursor ($*CH_2CH_2OH$). The dehydration of $*CH_2CH_2OH$ to ethylene (red line) is strongly exergonic across the whole range, explaining why ethylene dominates at moderate potentials. In contrast, the hydrogenation of $*CH_2CH_2OH$ to ethanol (green) and the vinyl-to-β-hydroxy-vinyl step (purple) become progressively more favourable with bias, predicting the eventual selectivity inversion toward ethanol beyond −1.8 V.
(c) Glyoxal C2 branch. Potential response of the glyoxal-centred hydrogenation ladder. The early *(CHO)CHOH → $*(CHO)CH_2OH$ and $*(CHO)CH_2OH$ → $*(CH_2OH)_2$ steps (red and green traces) are only weakly potential-dependent, remaining close to thermoneutral even at −2.0 V, which retards deep hydrogenation along this route. By contrast, the downstream *(CO)CHO → *(CHO)CHO hydrogenation (dark-blue) and the acetyl-derived $*CHCH_2$ → $*CH_2CH_2$ dehydration (light-blue) gain up to 1.5 eV of exergonicity over the same window, underscoring why the acetyl pathway contributes measurably to ethylene only at the most negative biases explored.
Collectively, the three panels reveal how unequal potential sensitivities among PCET steps steer product distributions: in the C1network they suppress methane at mild bias; in the vinyl branch they trigger an ethylene→ethanol switch near −1.8 V; and in the glyoxal/acetyl manifold they keep ethanol formation sluggish until the deepest cathodic regime.

Table S1. Cu-C bond distances (in Angstrom) for the different intermediates at -1.2V. The C-N distances are reported in brackets.

*CO_2	2.38	*CCHOH	1.90 (1.44)
*CO_2 bent	2.38	*CHCHOH	2.09
*HOCO	2.11	*$CHCH_2OH$	1.89
*CO	1.77	*CH_2CHOH	3.46
*CHO	1.92	*CH_2CH_2OH	2.27
*CHOH	1.84	*CH_3CH_2OH	3.56
*CH	1.94 (1.39)	*(CH)CO	1.98/1.90 (1.49/1.44)
*CH_2	1.95 (1.41)	*(CH_2)CO	2.31 (1.45/1.48)
*CH_3	2.17	*(CH_3)CO	1.92
*CH_4	3.34	*(CH_2)COH	1.94 (1.48/1.43)
*OCH_2	2.97	*(CH_3)CHO	3.31
*CH_2O	3.09	*(CH_3)CHOH	2.12
*OCH_3	2.33	*(CHO)CHO	3.69
*CH_3OH	3.29	*(CHO)CHOH	3.51
*CH_2OH	3.42	*(CHO)CH_2OH	3.35
*(CHO)CO	1.94	*(CHOH)CH_2OH	3.82
*(CHO)COH	2.00	*$(CH_2OH)_2$	3.46
*(CHOH)COH	2.09	*CCH	1.91/1.93 (1.45)
*CCH_2	1.98 (1.53/1.38)	*CHCH	1.90 (1.43)
*CH_2H_2	3.40	*$CHCH_2$	1.96 (1.50/1.42)

Table S2. GCP parameters for all investigated intermediates.

**System**	**a [V/e]**	**b [V]**	**c [eV]**	**C_{diff} [F]**	**PZC [V vs. SHE]**
Cu-MOF	0.1612960	-3.733774	-30331.43	3.09989	-0.92623
*CO_2	0.1625973	-3.474706	-33254.20	-3.07508	-1.18529
*CO_2 bent	0.1251544	-3.806234	-33253.61	-3.99507	-0.85377
*HOCO	0.1497261	-3.924300	-33253.33	-3.33943	-0.7357
*CO	0.0842745	-3.669624	-33269.66	-5.93299	-0.99038
*CHO	0.1073923	-3.753306	-32815.03	-4.65583	-0.90669
*CHOH	0.1006394	-3.714057	-32829.50	-4.96823	-0.94594

*CH	0.1485439	-3.916815	-32845.49	-3.36601	-0.74319
*CH_2	0.1076085	-3.556376	-32393.21	-4.64647	-1.10362
*CH_3	0.0928229	-3.654847	-32409.14	-5.3866	-1.00515
*CH_4	0.1216861	-3.581959	-32426.53	-4.10893	-1.07804
*(CH)CO	0.15734	-3.678639	-32984.41	-3.17783	-0.98136
*(CH_2)CO	0.120157	-3.565093	-32985.15	-4.16122	-1.09491
*(CH_2)COH	0.115108	-3.479548	-32999.68	-4.34375	-1.18045
*CCH_2	0.131229	-3.708863	-32545.41	-3.81013	-0.95114
*CH_2H_2	0.131408	-3.609673	-32583.32	-3.80494	-1.05033
*(CHO)CO	0.070587	-3.531669	-33422.73	-7.08346	-1.12833
*(CHO)COH	0.140613	-4.157215	-33422.19	-3.55586	-0.50279
*(CH)COH	0.143732	-3.677756	-32984.62	-3.4787	-0.98224
*CCH	0.099205	-3.690499	-33000.09	-5.04007	-0.9695
*CHCH	0.130775	-3.637045	-32547.33	-3.82336	-1.02296
*$CHCH_2$	0.120819	-3.487983	-32562.27	-4.13842	-1.17202
*(CHOH)COH	0.134868	-3.941867	-33437.90	-3.70733	-0.71813
*CCHOH	0.106615	-3.632075	-33454.12	-4.68977	-1.02793
*CH_2OH	0.116482	-3.588183	-32846.55	-4.29251	-1.07182
*CH_3OH	0.127060	-3.598696	-32863.43	-3.93515	-1.06130
*OCH_2	0.121199	-3.604722	-32831.62	-4.12545	-1.05528
*OCH_3	0.164431	-4.049638	-32847.00	-3.04079	-0.61036
*CH_2O	0.116459	-3.499552	-32832.04	-4.29336	-1.16045
*CHCHOH	0.157193	-4.028988	-33000.43	-3.18080	-0.63101
*CH_2CHOH	0.114142	-3.529671	-33018.35	-4.38051	-1.13033
*$CHCH_2OH$	0.104769	-3.765665	-33015.67	-4.77240	-0.89434
*CH_2CH_2OH	0.111338	-3.631578	-33035.60	-4.49083	-1.02842
*CH_3CH_2OH	0.123232	-3.633633	-33050.06	-4.05739	-1.02637
*(CH_3)CO	0.103008	-3.701554	-33001.66	-4.85399	-0.95845
*(CH_3)CHO	0.158570	-3.754692	-33018.53	-3.15318	-0.90531
*(CH_3)CHOH	0.095104	-3.628615	-33032.99	-5.25740	-1.03139
*(CHO)CHO	0.172890	-4.120347	-33424.14	-2.89201	-0.53965
*(CHO)CHOH	0.173533	-4.001947	-33439.62	-2.88130	-0.65805

*(CHO)CH_2OH	0.143745	-3.610370	-33455.61	-3.47838	-1.04963
*(CHOH)CH_2OH	0.166391	-3.758239	-33469.33	-3.00497	-0.90176
*$(CH_2OH)_2$	0.143081	-3.619980	-33487.08	-3.49452	-1.04002

Table S3. Microkinetic parameters for the C1 products.

Potential (V)	product	electrons	Ea(f) eV	Ea(b) eV	$k_{forward}$ (s^{-1})	$K_{backward}$ (s^{-1})	Net rate (s^{-1})	FE
-2	CH_2O	4	0	1.695	1	3.35E-29	1	0.153846
-2	CH_3OH	6	0	1.8771	2	2.39E-28	2	0.461538
-2	CH_4	8	0	2.78	1	1.99E-47	1	0.307692
-2	CO	2	0	3.38	1	1.65E-57	1	0.076923
-1.8	CH_2O	4	0	1.786	1	9.92E-31	1	0.2
-1.8	CH_3OH	6	0	2.4	1	2.10E-25	1	0.3
-1.8	CH_4	8	0	2.733	1	1.22E-46	1	0.4
-1.8	CO	2	0	3.45	1	1.10E-58	1	0.1
-1.6	CH_2O	4	0	1.93	1	3.78E-33	1	0.153846
-1.6	CH_3OH	6	0	1.952	2	8.43E-23	2	0.461538
-1.6	CH_4	8	0	2.7	1	4.39E-46	1	0.307692
-1.6	CO	2	0	3.37	1	2.43E-57	1	0.076923
-1.4	CH_2O	4	0	2.05	1	3.64E-35	1	0.153846
-1.4	CH_3OH	6	0	1.9275	2	1.82E-20	2	0.461538
-1.4	CH_4	8	0	2.65	1	3.03E-45	1	0.307692
-1.4	CO	2	0	3.132	1	2.43E-53	1	0.076923
-1.2	CH_2O	4	0	2.15	1	7.61E-37	1	0.153846
-1.2	CH_3OH	6	0	1.86605	2	1.44E-18	2	0.461538
-1.2	CH_4	8	0	2.6063	1	1.64E-44	1	0.307692
-1.2	CO	2	0	2.73	1	1.37E-46	1	0.076923
-1	CH_2O	4	0	2.23	1	3.45E-38	1	0.153846
-1	CH_3OH	6	0	1.758	2	5.91E-17	2	0.461538
-1	CH_4	8	0	2.56	1	9.86E-44	1	0.307692
-1	CO	2	0	2.17	1	3.51E-37	1	0.076923
-0.8	CH_2O	4	0	0.475	1	1.05E-08	1	0.153846
-0.8	CH_3OH	6	0	1.594	2	5.68E-15	2	0.461538
-0.8	CH_4	8	0	2.51	1	6.82E-43	1	0.307692
-0.8	CO	2	0	1.46	1	2.97E-25	1	0.076923

Table S4. Microkinetic parameters for the vinyl pathway C2 products.

Potential (V)	product	electrons	Ea(f) eV	Ea(b) eV	$k_{forward}$ (s^{-1})	$K_{backward}$ (s^{-1})	Net rate (s^{-1})	FE
-0.8	CH_3CH_2OH	12	0	2.516420752	1	5.32E-43	1	0.352941
-0.8	CH_2CH_2	12	0.47274	3.552739397	1.14E-08	2.07E-60	1.14E-08	1.35E-09
-0.8	CH_2CHOH	10	0	2.457322747	1	5.23E-42	1	0.294117

-0.8	CHCH	10	0	1.335977595	1	3.60E-23	1	0.352941
-1	CH_3CH_2OH	12	0	2.586057178	1	3.60E-44	1	0.352941
-1	CH_2CH_2	12	0.38178	3.681780403	3.86E-07	1.41E-62	3.86E-07	4.54E-08
-1	CH_2CHOH	10	0	2.459085598	1	4.89E-42	1	0.294117
-1	CHCH	10	0	1.393990332	1	3.82E-24	1	0.352941
-1.2	CH_3CH_2OH	12	0	2.667726123	1	1.53E-45	1	0.352940
-1.2	CH_2CH_2	12	0.28713	3.797130667	1.50E-05	1.63E-64	1.50E-05	1.77E-06
-1.2	CH_2CHOH	10	0	2.513567776	1	5.94E-43	1	0.294117
-1.2	CHCH	10	0	1.458441238	1	3.16E-25	1	0.352940
-1.4	CH_3CH_2OH	12	0	2.761427586	1	4.07E-47	1	0.352913
-1.4	CH_2CH_2	12	0.18860	3.898604353	0.000678	3.21E-66	0.000678	7.98E-05
-1.4	CH_2CHOH	10	0	2.620769282	1	9.40E-45	1	0.294094
-1.4	CHCH	10	0	1.529330315	1	2.03E-26	1	0.352913
-1.6	CH_3CH_2OH	12	0	2.867161567	1	6.82E-49	1	0.351478
-1.6	CH_2CH_2	12	0.08638	3.996387298	0.03538	7.31E-68	0.03538	0.004145
-1.6	CH_2CHOH	10	0	2.780690114	1	1.93E-47	1	0.292898
-1.6	CHCH	10	0	1.60665756	1	1.02E-27	1	0.351478
-1.8	CH_3CH_2OH	12	0	2.984928067	1	7.17E-51	1	0.315789
-1.8	CH_2CH_2	12	0	4.080417556	1	2.83E-69	1	0.105263
-1.8	CH_2CHOH	10	0	2.993330274	1	5.18E-51	1	0.263157
-1.8	CHCH	10	0	1.729330315	1	8.88E-30	1	0.315789
-2	CH_3CH_2OH	12	0	3.114727085	1	4.73E-53	1	0.315789
-2	CH_2CH_2	12	0	4.140695127	1	2.75E-70	1	0.105263
-2	CH_2CHOH	10	0	3.25868976	1	1.80E-55	1	0.263157
-2	CHCH	10	0	1.782702141	1	1.13E-30	1	0.315789

Table S5. Microkinetic parameters for the acetaldehyde pathway C2 products.

Potential (V)	product	electrons	Ea(f) eV	Ea(b) eV	$k_{forward}$ (s^{-1})	$K_{backward}$ (s^{-1})	Net rate (s^{-1})	FE
-0.8	CH_3CH_2OH	12	0	2.516420752	1	5.32E-43	1	0.749999
-0.8	CH_2CH_2	12	0.472739	3.552739397	1.14E-08	2.07E-60	1.14E-08	2.86E-09
-0.8	CH_3CHO	10	0	3.451854618	1	1.03E-58	1	0.249999
-1	CH_3CH_2OH	12	0	2.586057178	1	3.60E-44	1	0.749999
-1	CH_2CH_2	12	0.381780	3.681780403	3.86E-07	1.41E-62	3.86E-07	9.65E-08
-1	CH_3CHO	10	0	3.636503803	1	8.12E-62	1	0.249999
-1.2	CH_3CH_2OH	12	0	2.667726123	1	1.53E-45	1	0.749997
-1.2	CH_2CH_2	12	0.287130	3.797130667	1.50E-05	1.63E-64	1.50E-05	3.75E-06
-1.2	CH_3CHO	10	0	3.790084876	1	2.13E-64	1	0.249999
-1.4	CH_3CH_2OH	12	0	2.761427586	1	4.07E-47	1	0.749872
-1.4	CH_2CH_2	12	0.188604	3.898604353	0.00067	3.21E-66	0.000678	0.000169
-1.4	CH_3CHO	10	0	3.91259784	1	1.87E-66	1	0.249957
-1.6	CH_3CH_2OH	12	0	2.867161567	1	6.82E-49	1	0.743424
-1.6	CH_2CH_2	12	0.086388	3.996387298	0.03537	7.31E-68	0.035379	0.008767
-1.6	CH_3CHO	10	0	4.004042693	1	5.43E-68	1	0.247808
-1.8	CH_3CH_2OH	12	0	2.984928067	1	7.17E-51	1	0.6
-1.8	CH_2CH_2	12	0	4.080417556	1	2.83E-69	1	0.2
-1.8	CH_3CHO	10	0	4.064419436	1	5.26E-69	1	0.2

-2	CH_3CH_2OH	12	0	3.114727085	1	4.73E-53	1	0.6
-2	CH_2CH_2	12	0	4.140695127	1	2.75E-70	1	0.2
-2	CH_3CHO	10	0	4.093728069	1	1.69E-69	1	0.2

Table S6. Microkinetic parameters for the glyoxal pathway C2 products.

Potential (V)	product	electrons	Ea(f) eV	Ea(b) eV	$k_{forward}$ (s^{-1})	$K_{backward}$ (s^{-1})	Net rate (s^{-1})	FE
-0.8	(CHO)CHO	6	0	5.16974985	1	1.42E-87	1	0.222222
-0.8	(CHO)CH_2OH	8	0	3.45185461	1	1.03E-58	1	0.111111
-0.8	$(CH_2OH)_2$	10	0	1.78219638	1	1.15E-30	1	0.333333
-0.8	CH_3CH_2OH	12	0	2.51642075	1	5.32E-43	1	0.333333
-0.8	CH_2CH_2	12	0.472739	3.55273939	1.14E-08	2.07E-60	1.14E-08	1.27E-09
-1	(CHO)CHO	6	0	5.60807103	1	6.15E-95	1	0.222222
-1	(CHO)CH_2OH	8	0	3.63650380	1	8.12E-62	1	0.111111
-1	$(CH_2OH)_2$	10	0	1.83784910	1	1.34E-31	1	0.333333
-1	CH_3CH_2OH	12	0	2.58605717	1	3.60E-44	1	0.333333
-1	CH_2CH_2	12	0.381780	3.68178040	3.86E-07	1.41E-62	3.86E-07	4.29E-08
-1.2	(CHO)CHO	6	0	6.05813663	1	1.7E-102	1	0.222221
-1.2	(CHO)CH_2OH	8	0	3.79008487	1	2.13E-64	1	0.111110
-1.2	$(CH_2OH)_2$	10	0	1.89888915	1	1.26E-32	1	0.333332
-1.2	CH_3CH_2OH	12	0	2.66772612	1	1.53E-45	1	0.333332
-1.2	CH_2CH_2	12	0.287130	3.79713066	1.50E-05	1.63E-64	1.50E-05	1.67E-06
-1.4	(CHO)CHO	8	0	6.51994663	1	2.9E-110	1	0.222205
-1.4	(CHO)CH_2OH	4	0	3.91259784	1	1.87E-66	1	0.111102
-1.4	$(CH_2OH)_2$	12	0	1.96531654	1	9.64E-34	1	0.333308
-1.4	CH_3CH_2OH	12	0	2.76142758	1	4.07E-47	1	0.333308
-1.4	CH_2CH_2	4	0.188604	3.89860435	0.000678	3.21E-66	0.000678	7.54E-05
-1.6	(CHO)CHO	8	0	6.99350103	1	3.3E-118	1	0.221352
-1.6	(CHO)CH_2OH	4	0	4.00404269	1	5.43E-68	1	0.110676
-1.6	$(CH_2OH)_2$	12	0	2.03713127	1	5.99E-35	1	0.332028
-1.6	CH_3CH_2OH	12	0	2.86716156	1	6.82E-49	1	0.332028
-1.6	CH_2CH_2	4	0.086387	3.99638729	0.035379	7.31E-68	0.035379	0.003915
-1.8	(CHO)CHO	8	0	7.47879985	1	2.3E-126	1	0.2
-1.8	(CHO)CH_2OH	4	0	4.06441943	1	5.26E-69	1	0.1
-1.8	$(CH_2OH)_2$	12	0	2.11433334	1	3.03E-36	1	0.3
-1.8	CH_3CH_2OH	12	0	2.98492806	1	7.17E-51	1	0.3
-1.8	CH_2CH_2	4	0	4.08041755	1	2.83E-69	1	0.1
-2	(CHO)CHO	8	0	7.97584307	1	1.0E-134	1	0.2
-2	(CHO)CH_2OH	4	0	4.09372806	1	1.69E-69	1	0.1
-2	$(CH_2OH)_2$	12	0	2.19692274	1	1.24E-37	1	0.3
-2	CH_3CH_2OH	12	0	3.11472708	1	4.73E-53	1	0.3
-2	CH_2CH_2	4	0	4.14069512	1	2.75E-70	1	0.1

Table S7. α-check considering the extreme values of 0.3 and 0.7 at an applied bias of -1.2 V for the TS of the whole CO2RR mechanism. The 0.456 value is the one reported in the main text. Only intermediates with a TS are reported. The RDS for each pathways are reported in red.

	α = 0.3	α = 0.456	α = 0.7
**$CO_2 \rightarrow CO_2$Tilt**	0.27	0.23	0.16
**CO_2Tilt $\rightarrow$ HOCO**	0.40	0.42	0.46
**CO $\rightarrow$ CHO**	0.24	0.18	0.09
**CHO $\rightarrow$ CHOH**	0.45	0.50	0.58
**$OCH_2 \rightarrow CH_2OH$**	0.28	0.24	0.18
**$OCH_3 \rightarrow CH_3OH$**	0.30	0.27	0.22
**COCH $\rightarrow$ COHCH**	0.48	0.55	0.65
**CHCH $\rightarrow$ $CHCH_2$**	0.35	0.34	0.34
**CCH $\rightarrow$ CCH_2**	0.44	0.48	0.55
**COHCHO $\rightarrow$ COHCHOH**	0.30	0.28	0.23
**CHCHOH $\rightarrow$ $CHCH_2OH$**	0.30	0.27	0.22
**$CH_2CH_2OH \rightarrow CH_2CH_2$**	0.89	0.89	0.89
**$CH_3CHO \rightarrow CH_3CHOH$**	0.47	0.53	0.63
**CHOCHOH $\rightarrow$ $CHOCH_2OH$**	0.29	0.26	0.21
**HER**	0.71	0.89	1.17
